# Supplementary material for: β-hydroxy-β-methylbutyrate-enriched nutritional supplements for obese adults during weight loss: study protocol of a randomised, double-blind, placebo-controlled clinical trial
Source: BMJ Open. 2022 Jun 22;12(6):e055420. doi: 10.1136/bmjopen-2021-055420 (PMC9226916; doi:10.1136/bmjopen-2021-055420)
Supplement: Author's manuscript [file bmjopen-2021-055420.draft_revisions.pdf]

BMJ Open

BMJ Open is committed to open peer review. As part of this commitment we make the peer review history of every article we publish publicly available.

When an article is published we post the peer reviewers' comments and the authors' responses online. We also post the versions of the paper that were used during peer review. These are the versions that the peer review comments apply to.

The versions of the paper that follow are the versions that were submitted during the peer review process. They are not the versions of record or the final published versions. They should not be cited or distributed as the published version of this manuscript.

BMJ Open is an open access journal and the full, final, typeset and author-corrected version of record of the manuscript is available on our site with no access controls, subscription charges or pay-per-view fees (http://bmjopen.bmj.com).

If you have any questions on BMJ Open's open peer review process please email info.bmjopen@bmj.com

BMJ Open

β-hydroxy-β-methylbutyrate-enriched nutritional supplements for obesity adults during weight loss: study protocol of a randomized double-blind placebo-controlled clinical trial

Journal:	*BMJ Open*
Manuscript ID	bmjopen-2021-055420
Article Type:	Protocol
Date Submitted by the Author:	12-Jul-2021
Complete List of Authors:	Jing, Xiaofan; Sichuan University West China Hospital, Department of Clinical Nutrition<br>Liang, Yuxiang; Sichuan University West China Hospital<br>Wang, Renjie; Sichuan University<br>Fu, Hongbo; Sichuan University<br>Jiang, Jiaojiao; Sichuan University<br>Yang, Ming; Sichuan University; Sichuan University
Keywords:	Nutritional support < GASTROENTEROLOGY, NUTRITION & DIETETICS, Nutrition < TROPICAL MEDICINE, Nutritional support < ONCOLOGY, Musculoskeletal disorders < ORTHOPAEDIC & TRAUMA SURGERY

SCHOLARONE™
Manuscripts

β-hydroxy-β-methylbutyrate-enriched nutritional supplements for obesity adults during weight loss: study protocol of a randomized double-blind placebo-controlled clinical trial

Xiaofan Jing [1]; Yuxiang Liang [2]; Renjie Wang [2]; Hongbo Fu [3]; Jiaojiao Jiang [2, *]; Ming Yang [3,4, *]

1. Department of Clinical Nutrition, West China Hospital, Sichuan University, Chengdu, China

2. Rehabilitation Center, West China Hospital, Sichuan University, Chengdu, China

3. National Clinical Research Center for Geriatrics, West China Hospital, Sichuan University, Chengdu, China

4. Center of Gerontology and Geriatrics, West China Hospital, Sichuan University, Chengdu, China

***Corresponding authors:**

Jiaojiao Jiang

Rehabilitation Center, West China Hospital, Sichuan University, No 37 Guoxue Lane, Chengdu 610041, Sichuan, China

Tel: +86 28 8542 2313

Fax: +86 28 8542 2313

Email: jiangjiaojiao1997@163.com

Ming Yang

National Clinical Research Center for Geriatrics, West China Hospital, Sichuan University, No 37 Guoxue Lane, Chengdu 610041, Sichuan, China

Tel: +86 28 8542 2326

Fax: +86 28 8542 2321

Email: yangmier@gmail.com; yangmier@scu.edu.cn

Keywords: sarcopenia; muscle wasting; HMB; obesity; oral nutritional supplements

Word Count: 3586 words

For peer review only

For peer review only - http://bmjopen.bmj.com/site/about/guidelines.xhtml

Abstract

Introduction Obesity is a public health challenge worldwide. To maintain a healthy weight, dieting and lifestyle changes are the first-line interventions; however, these interventions are of poor compliance and may cause body composition changes, mainly skeletal muscle wasting (sarcopenia). Dietary supplements for improving body composition while inducing weight loss are therefore needed. β-hydroxy-β-methylbutyrate (HMB) has been proven to be effective for improving muscle mass and muscle strength in athletes, older adults, and cancer patients. We aim to evaluate the effectiveness and safety of HMB-enriched nutritional supplements for improving muscle mass and muscle function in obese adults during calorie restriction.

Method and analysis A total of 72 Chinese adults with obesity will be randomized to receive HMB-enriched nutritional supplements (65 g/day) or a placebo for 12 weeks. Participants in both groups will also receive caloric restriction based on the individualized nutrition guidance of dieticians. Participants and investigators will be blinded to the allocations. The primary outcome will be the mean change in whole-body skeletal muscle mass. The secondary outcomes include the mean change of appendicular skeletal muscle mass, body fat mass, basal metabolic rate, phase angle, muscle function, and serum biomarkers. Enrollment will commence July 2021 and proceeds until December 2021.

Ethics and dissemination This protocol has been approved by the Biomedical Ethics Committee of West China Hospital (2021-771). All potential subjects will be required to sign a written informed consent. The results of this study will be reported in peer-reviewed academic journals and conferences.

Trial registration number NCT04953936

For peer review only - http://bmjopen.bmj.com/site/about/guidelines.xhtml

INTRODUCTION

Obesity is a chronic disease featured by abnormal or excess body fat that impairs health. [1] Over the past three decades, obesity has become a common health problem that plagues modern people worldwide [2]. For example, more than one-third (36.5%) of the US adult population is obese defined by a body mass index (BMI) ≥ 30 kg/m^2 [3]. In China, the prevalence of abdominal obesity among adults was approximately 29%, and the number of obese adults is approximately 277.8 million [4]. Obesity is not only a risk factor for cardiovascular diseases, diabetes, dyslipidemia, chronic kidney disease, and some types of cancer, but also increases the risk of psychological disorders, disability, poor quality of life, and death [1 2 5 6]. Therefore, obesity prevention and control have become one of the key contemporary public health challenges [7].

It is well known that increasing physical activity and improving dietary strategies are the keys to the prevention and management of obesity [1]. Many dietary strategies have been proposed for weight loss, such as very low energy diets, ketogenic diets, high protein diets, and high fiber diets. However, individuals who apply these approaches may experience weight loss accompanied by body composition changes, mainly skeletal muscle wasting (sarcopenia) [3]. Sarcopenia may result in a lower resting metabolic rate and a greater tendency to regain body fat. Recently, there is growing evidence that certain nutritional fortifications can improve body composition during weight loss [8]. A variety of over-the-counter dietary supplements for this purpose are available, and they have gained popularity among consumers for weight management [9].

β-hydroxy-β-methylbutyrate (HMB) is the active metabolite of leucine, which regulates muscle protein synthesis, and HMB plays an important role in this process. However, the rate of conversion of leucine

For peer review only - http://bmjopen.bmj.com/site/about/guidelines.xhtml

to HMB is very low, and only approximately 5% of leucine in humans is converted to HMB. Studies have found that HMB has the effect of increasing muscle mass and reducing exercise injury, and its mechanism of action includes both inhibitions of proteolysis and the promotion of protein synthesis. The good tolerability and safety of HMB in humans have been well established [10-12]. As early as 1995, the US Food and Drug Administration (FDA) has listed HMB in "Generally Recognized as Safe" (GRAS) ingredients. In 2010, China's former Ministry of Health announced the approval of HMB as a "new resource food".

Studies have shown that HMB supplementation is also beneficial in building muscle strength and may be beneficial in improving body composition. For example, a systematic evaluation that included 7 RCTs showed that HMB supplementation helped maintain muscle mass in older adults and prevent muscle atrophy due to bed rest or other factors [13]. Another systematic evaluation that included 9 RCTs also showed that HMB supplementation helped improve body composition in older adults [14]. Daily HMB supplementation was also reported to increase the benefits of exercise and improves body composition in healthy adults in the early stages of regular exercise [15]. Therefore, HMB is expected to be a muscle-protective nutritional fortification that can effectively improve muscle mass and muscle strength in both healthy adults and the elderly. However, evidence regarding the effectiveness of HMB on maintaining skeletal muscle in obese adults during weight loss was limited in the literature. Additionally, very few clinical trials regarding HMB have been conducted in Chinese populations. Therefore, this trial aims to assess the effectiveness and safety of HMB-enriched nutritional supplements for improving muscle mass and muscle function in Chinese adults with obesity during the weight loss process using calorie restriction.

For peer review only - http://bmjopen.bmj.com/site/about/guidelines.xhtml

METHODS AND ANALYSIS

Study Design

We designed a single-center, randomized, parallel-group, double-blind, placebo-controlled trial. We plan to recruit 72 Chinese obese adults who intend to perform weight loss via calorie restriction. The participates will be randomly assigned to either the intervention group and the control group at a 1:1 ratio and will receive HMB-enriched nutritional supplements or placebo, respectively, for 12 weeks. The flow chart of this trial is presented in Figure 1 while the overall schedule of this trial is shown in Table 1.

Recruitment

This trial will be conducted at West China Hospital, Sichuan University, China. To reach the target sample size, we plan to advertise this trial using the hospital's homepage, WeChat, and flyers inside and outside the hospitals.

Participants

Inclusion Criteria

Individuals who meet all the following criteria will be invited to participate in this study: (1) men and women aged 30-50 years; (2) currently having obesity defined by body mass index (BMI) ≥ 28 kg/m^2 (based on measured body weight and height at booking, using validated scales) [16]; (3) having an intention to lost weight via caloric restriction; (4) healthy and able to walk independently; (5) able to eat independently to meet their energy needs; (6) without significant body weight change in the last 6

For peer review only - http://bmjopen.bmj.com/site/about/guidelines.xhtml

month (less than 5% change in body weight); (7) having sedentary habit defined by Sedentary Behavior Research Network [17]; (8) able to collaborate with the research staff. Individuals will be recruited by research nurses or physicians. The study protocol will be provided in advance and individuals who wish to participate will be offered the opportunity to discuss the study and consent to participate. All participants will be required to sign the written informed consent before participation.

Exclusion Criteria

Individuals with any of the following criteria will be excluded: (1) a history of intolerance to enteral nutrition, food (e.g., lactose intolerance), or being allergic to the components of the HMB-enriched nutritional supplements (e.g., soy or corn); (2) having any implants (e.g., pacemakers); (3) current use of other nutritional supplements or drugs that may have an impact on the effectiveness of the intervention (e.g., glucocorticoids, antineoplastic drugs, antituberculosis drugs, sedatives, antipsychotics, muscle relaxants, monoamine oxidase inhibitors, antibiotics) within 3 months prior to enrollment; (4) secondary obesity caused by diseases (e.g., hypothalamic diseases and hypercortisolism) or drugs (e.g. glucocorticoids, insulin, tricyclic antidepressants); (5) clinically visible edema; (6) trauma, surgery, hospitalization, fall, or fracture within 6 months prior to enrollment; (7) pregnancy, having pregnancy plans, or lactation; (8) individuals who are participating in other clinical trials; any acute illness (e.g., acute infection, myocardial infarction, arrhythmia, myocarditis, appendicitis, etc.); (9) swallowing disorders; abnormal thyroid function (including hyperthyroidism and hypothyroidism); (10) history of diabetes, respiratory diseases, cardiovascular diseases, uncontrolled hypertension, kidney diseases, digestive system diseases, renal insufficiency, mental illness, neurological diseases, hematologic diseases, liver diseases (except for fatty liver), chronic infection (e.g., tuberculosis),

For peer review only

For peer review only - http://bmjopen.bmj.com/site/about/guidelines.xhtml

immune system diseases, joint disorders, any type of tumor; (11) alcohol consumption over two standard alcoholic beverages (20 g of alcohol/day); (12) difficulty to comply with the study protocol; and (13) other conditions that indicate the individuals are inappropriate for participation in this study.

Withdrawal criteria

We will withdraw participants from this trial after randomization for the following reasons: (1) if the participants request to withdraw from this trial; (2) if there is evidence of a complication or the occurrence of an unknown disease, the investigators can decide that the patient cannot continue in the study; (3) if there is an adverse event classified as grade 3 or higher according to the Common Terminology Criteria for Adverse Events (CTCAE) version 5.0; (4) if investigators believe the participants are unsuitable for continuing participating in the study for any other reason.

Randomization and allocation concealment

We will use the stratified blocked randomization method to perform randomization. Random number sequence was automatically generated by R version 4.0.3 (R Foundation for Statistical Computing, Vienna, Austria) before the inclusion of the first participants. Gender was first used as a stratification factor, and 36 cases of each sex were included, and then block group randomization was performed for each sex, with block group lengths set to 2-8 and randomly selected by the computer.

To perform allocation concealment, we will seal the randomization list in sequentially numbered opaque envelopes. The envelopes were opened sequentially at the beginning of the intervention in the order of entry, and the grouping of participants was determined according to the allocation scheme inside the envelope.

For peer review only

For peer review only - http://bmjopen.bmj.com/site/about/guidelines.xhtml

Blinding

By using identical doses and appearance of HMB-enriched nutritional supplements and placebos, participants and outcome assessors will be blind to group allocation.

Interventions

The participants will be randomly assigned into two groups: the intervention group (Group A) and the control group (Group B). The participants in both groups will perform a caloric restriction diet. They will receive individualized nutritional guidance from professional dietitians by video regarding caloric restriction strategies. The principle for caloric restriction will be based on the equation: energy intake = ideal body weight (i.e., height (cm) -105) × 25 kcal/kg/d [18]. The compliance will be followed via a WeChat group and telephone. The intervention duration in both groups will be 12 weeks.

Group A

The participants in Group A will receive oral HMB-enriched nutritional supplements (65 g, once daily), which include soybean isolate, protein, whey protein, casein, HMB, L-glutamine, wheat oligopeptide, medium-chain fatty acids, vitamin D, vitamin E, konjac powder, and fish oil powder.

Group B

The participants in Group B will receive a placebo (maltodextrin 65 g once daily) with the same package as the intervention.

Outcomes

As shown in Table 1, the assessors, who were blinded to the group allocation, will ask the participants

For peer review only - http://bmjopen.bmj.com/site/about/guidelines.xhtml

to complete questionnaires (including demographic characteristics, physical activity, and energy intake), anthropometric measurement (including body weight, height, waist circumference, and hip circumference), heart rate, blood pressure, handgrip strength measurement, sit-to-stand test, and body composition at the baseline, the 6-week, and the end of the 12-week intervention. Additionally, participates will complete self-reported questionnaires at the 3-week and the 9-week.

Moreover, fasting blood will be taken by a nurse at baseline and the end of 12-week intervention after intervention, for laboratory analysis of biomarkers of glucose metabolism (fasting glucose and insulin), albumin, hemoglobin, alanine aminotransferase (ALT), aspartate aminotransferase (AST), gamma-glutamyl transpeptidase (GGT), Cystatin C, creatinine, estimated glomerular filtration rate (eGFR), lipid profiles, and full blood counts. The fasting blood will be sent to the clinical laboratory of West China Hospital, Sichuan University within 2 h after withdrawal. These biomarkers will be tested using standard methods. Moreover, Homeostasis Model Assessment of Insulin Resistance (HOMA-IR) was calculated using the equation: fasting glucose (mg/dL) × fasting insulin (μIU/mL)/405.

Primary outcome

The mean change in whole-body skeletal muscle mass (SMM) from the beginning to the end of the 12-week intervention will be measured for all participants. The whole-body lean body mass will be measured by trained research staff using a bioimpedance analysis device (BIA; InBody 770; Biospace Co., Seoul, Korea). The participants will be guided through the BIA test. They will be asked to stand right at least 5 m before testing, then to step on the footplate barefoot with their heels on the rear sole electrodes and their hand holding the hand electrode. Their arms should be straight and should not touch the side of the body during the test.

For peer review only - http://bmjopen.bmj.com/site/about/guidelines.xhtml

Secondary outcomes

We will measure the following secondary outcomes:

- The mean change in appendicular skeletal muscle mass (ASMM), trunk skeletal muscle mass (TSMM), total body fat mass (BFM), appendicular body fat mass (ABFM), trunk body fat mass (TBFM), basal metabolic rate (BMR), phase angle (PA), and body weight (BW) from the beginning to weeks 6 and 12 will be measured for all participants. These data will be measured using InBody 770 (Biospace Co., Seoul, Korea).
- The mean change in handgrip strength (HGS) from the beginning to weeks 6 and 12 will be measured for all participants. A trained nurse will measure HGS using a digital handheld dynamometer (EH101; Xiangshan Inc, Guangdong, China) to the nearest 0.1 kg according to the recommendation of the Chinese National Physical Fitness Evaluation Standard [19]. The participants will be asked to perform three measurements for each hand, with rest periods of ≥ 30 s between each measurement. The HGS was determined as the maximum value of the six records.
- The mean change in waist circumference and hip circumference from the beginning to weeks 6 and 12 will be measured for all participants. A trained nurse will measure waist circumference using a tape at the level midway between the lower rib margin and the iliac crest with the participant breathing out gently. Hip circumference was measured as the maximum circumference over the buttocks [20].
- The mean change in physical activity from the beginning to weeks 3, 6, 9, and 12 will be measured for all participants using the self-reported International Physical Activity Questionnaire Short Form (IPAQ-SF) [21].

For peer review only - http://bmjopen.bmj.com/site/about/guidelines.xhtml

- The mean change in the score of the sit-to-stand test from the beginning to weeks 6 and 12 will be measured for all participants. A trained nurse will perform the 30-second sit-to-stand test [22], which is a classic test for measuring the performance of lower extremity muscles.
- The mean change in energy intake from the beginning to weeks 3, 6, 9, and 12will be measured for all participants using the self-reported Food Frequency Questionnaire (FFQ) [23].
- The mean change of lipid profiles, fasting glucose, fasting insulin, HOMA-IR, and albumin from the beginning to the end of the 12-week intervention will be measured for all participants.

Safety outcomes

We will record all adverse events during the entire study period. The participants will be asked to complete a self-reported questionnaire regarding any adverse event every 3 weeks. The participants will also encourage to report adverse events to the research staff via telephone when there is any advent event occurrence. We will rank the severity of adverse events according to the Common Terminology Criteria for Adverse Events (CTCAE) version 5.0. The cause of the adverse events will be ranked as unknown, definitely not related, probably not related, possibly related, probably related, definitely related [24].

Moreover, we will report the mean change in the laboratory tests regarding adverse events from the beginning to the end of the 12-week intervention: hemoglobin, AST, ALT, GGT, Cystatin C, creatinine, and eGFR.

Feasibility outcomes

We will record the recruitment rate and completion rate to assess the feasibility of this trial.

For peer review only - http://bmjopen.bmj.com/site/about/guidelines.xhtml

Additionally, the adherence rate to the interventions will be calculated. Moreover, we will record the reasons for participants' ineligibility and for dropping out of this trial.

Data Collection, Management, and Monitoring

Research coordinators will formally obtain the participants' informed consent before collecting and recording data in the case report form (CRF). The assessment of outcomes will be done by assessors who are unaware of the assignment of the groups.

To ensure that participants adhere to the research strategy, all participants will be included in the WeChat group and the investigators will call them at least one day beforehand for each appointment and follow-up session. Participants who do not attend follow-up sessions will be asked for their reasons and telephone contacts will be made to encourage adherence to the intervention and attendance at appointments. We will also contact participants via WeChat or telephone once a week to determine if any AEs have occurred.

For the study's quality, monitoring and auditing of the data will be carried out. Prior to the beginning of the study, there will be an introductory meeting. At every important point in the trial, such as participant enrolment, the midway point, and the conclusion, monitoring staff will visit the institutions. In addition, monitoring staff will ensure consistency in the documentation of data contained in the CRF and source document, as well as ensuring that the entire study process is conducted according to the protocol.

Sample Size Estimation

To date, no studies have been done to determine whether HMB-enriched nutritional supplements are

For peer review only

For peer review only - http://bmjopen.bmj.com/site/about/guidelines.xhtml

effective and safe for improving muscle mass and muscle function in obese adults during weight loss. To determine the sample size, we examined similar studies on HMB and referred to the study by Peng et al. that evaluated the effectiveness of HMB for improving skeletal muscle mass and quality in pre-frail older adults [25]. Using a 0.05 significance level, 80% statistical power, and 25% attrition rate, a sample size of 70 was assumed using an assumed effective size of 0.93. Moreover, to achieve a gender-balanced distribution within and between groups, a random stratification method including 36 samples per district is proposed, adding up to 72 samples in total.

Statistical Analysis Plan

The statistical analysis will be performed by an independent statistical expert blinded to the group allocation. SPSS version 26.0 (IBM Corp, Armonk, NY) and R version 4.0.3 (R Foundation for Statistical Computing, Vienna, Austria) will be used for statistical analysis.

Each group will be summarized in terms of demographic characteristics and baseline variables. For comparisons of continuous variables, two-sample T-tests or Mann–Whitney U tests will be used, whereas for categorical variables, the χ^2 test or Fisher's exact test will be used.

As aforementioned, the primary outcome will be in SMM from the beginning to the end of the 12-week intervention. The independent two-sample T-test or Mann-Whitney U-test will be used based on the distributional normality. We will make adjustments based on the covariates if there are significant demographic differences between the groups or if baseline variables differ significantly. In the primary analysis, the full analysis set (FAS) will be the main data set. Participants initially assigned to either group will be included in the FAS. However, our analysis will not use the data of illegible participants, subjects without any intervention, or subjects without any outcome measurement. For sensitivity

For peer review only - http://bmjopen.bmj.com/site/about/guidelines.xhtml

analysis, we will use a protocol-specific set to compare with the results from the FAS. A per-protocol analysis will include participants who achieved the completion of the study without major deviations from protocol, who provided all outcomes, and who received at least 75% of the planned intervention. A significance level of two-sided $p < 0.05$ will be used. If needed, the multiple imputation method will be adopted for missing data.

We will use the same methods for analyzing secondary outcomes as we did for analyzing the primary outcome. A paired t-test or Wilcoxon signed-rank tests will be used to assess the changes in outcome measures within groups before and after the intervention. To validate trend differences per visit, we will use repeated measures analysis of variance with post hoc tests.

Moreover, subgroup analysis will be done to identify whether sex or Asian Working Group on Sarcopenia (AWGS)-defined sarcopenia [26] at baseline affect the clinical response to HMB-enriched nutritional supplements.

Ethics and Dissemination

This trial has been registered in ClinicalTrials.gov (reference NCT04953936). This trial will be conducted following the Declaration of Helsinki [27]. This study protocol was drafted according to Standard Protocol Items: Recommendations for Interventional Trials (SPIRIT) statement [28]. It has been approved by the Biomedical Ethics Committee of West China Hospital (reference 2021-771). Any protocol amendments will be reapproved by the Biomedical Ethics Committee of West China Hospital and will be reflected in the participants' informed consent form. We will obtain signed consent forms from all participants regarding the modification.

We intend to publish the results of this trial in appropriate academic peer-reviewed journals and

For peer review only - http://bmjopen.bmj.com/site/about/guidelines.xhtml

conferences.

Patient and Public Involvement

We did not involve patients or the public in the planning and design of this study.

DISCUSSION

This study will be a double-blind, randomized, parallel-group, placebo-controlled trial aiming to explore the effectiveness and safety of HMB-enriched nutritional supplements for improving muscle mass and muscle strength in obese adults during the weight loss process using calorie restriction. It will determine the feasibility of future large-scale clinical trials on HMB-based interventions for improving body composition (especially muscle health) in obese adults when performing weight loss plans.

The effectiveness of HMB for improving muscle mass and muscle strength has been well studied in athletes, healthy adults, older adults, and cancer patients [29]. However, we found only two clinical trials addressing the effectiveness of HMB on muscle health in obese adults [30 31]. In 2019, a randomized controlled trial (RCT) found that supplementing with HMB for 6 weeks reduced weight, waist, and abdominal circumference, and even increased strength without resistance training, and without affecting lean mass, in sedentary and overweight women [30]. Most recently, Rondanelli et al. [31] reported that using an oral nutrition supplement containing arginine, glutamine, and HMB for four weeks, it was observed that visceral adipose tissue and BFM were statistically significantly reduced, whereas fat-free mass (mainly muscle mass) remained the same.

Our trial has some strengths. First, it will be the first to evaluate the effectiveness of HMB-enriched

For peer review only - http://bmjopen.bmj.com/site/about/guidelines.xhtml

nutritional supplements for improving muscle health in Chinese obese adults. Second, we will use a double-blinded design to minimize bias due to demand characteristics, the placebo effect, and measurement bias. Last, we will focus on not only muscle mass and fat mass but also muscle function (i.e., handgrip strength and physical performance).

This trial also has some limitations. First, this will be a single-center trial and the sample size is relatively small. Therefore, the representativeness of our study sample might be limited. Second, participants in this study may be heterogeneous, given the wide age range. This heterogeneity should be considered when interpreting the results. Third, it might not be possible to completely control the dietary and physical activity of all participants. However, we will record the physical activity and energy intakes and will adjust for them as potential confounders. Fourth, the muscle mass and fat mass will be measured with a BIA device rather than a dual-energy X-ray absorptiometry (DXA) scanner. However, it was recently shown that DXA and segmental multifrequency BIA (InBody 770, which we will use in this trial) were equally accurate for estimating muscle mass and fat mass [32].

Trial status

This protocol is version 1.1, written on May 17, 2021. The recruitment will be conducted from July 2021 to December 2021.

Author contributions

Conceptualization, M.Y.; methodology, M.Y., J.J., and X.J.; validation, M.Y., and J.J.; formal analysis, J.J., and X.J.; investigation, X.J., H.F., R.W., and Y.L.; resources, M.Y.; data curation, J.J.; writing-original draft preparation, X.J., and M.Y.; writing-review and editing, J.J.; visualization, X.J.;

For peer review only - http://bmjopen.bmj.com/site/about/guidelines.xhtml

supervision, M.Y.; project administration, J.J.; funding acquisition, M.Y. All authors have read and agreed to the published version of the manuscript.

Article Summary

Strengths and limitations

- This is the first randomised, double-blind, placebo-controlled trial to explore the possible effectiveness and safety of HMB-enriched nutrition supplements for maintaining skeletal muscle mass and function in obese adults during calorie restriction.
- Using identical doses and appearance of HMB-enriched nutritional supplements and placebos to mask participants and outcome assessors.
- Hydration status of the participants may affect the results of bioimpedance analysis.
- The sample size of this trial is relatively small.

Funding

This trial was supported by the National Key R&D Program of China (No. 2018YFC2002104). The sponsor had no role in the design of this trial and will not take part in the methods, data collection, and data analysis of this trial.

Competing interests

The authors declare no conflict of interest.

For peer review only - http://bmjopen.bmj.com/site/about/guidelines.xhtml

Acknowledgements

None.

Patients consent for publication

Not required.

Data availability statement

The data of this trial will be available from the corresponding author on reasonable request.

Ethics approval

This study protocol has been approved by the Biomedical Ethics Committee of West China Hospital (reference 2021-771). This study protocol has been registered in ClinicalTrials.gov (reference NCT04953936).

Open access

This is an open access article distributed in accordance with the Creative Commons Attribution Non Commercial (CC BY-NC 4.0) license, which permits others to distribute, remix, adapt, build upon this work non-commercially, and license their derivative works on different terms, provided the original work is properly cited, appropriate credit is given, any changes made indicated, and the use is non-commercial. See: http://creativecommons.org/licenses/by-nc/4.0/.

For peer review only

ORCID ID

Ming Yang https://orcid.org/0000-0002-1875-1692

References

1. Wharton S, Lau DCW, Vallis M, et al. Obesity in adults: a clinical practice guideline. *Cmaj* 2020;192(31):E875-E91. doi: 10.1503/cmaj.191707 PMID - 32753461
2. Gregg EW, Shaw JE. Global Health Effects of Overweight and Obesity. *New Engl J Medicine* 2017;377(1):80-81. doi: 10.1056/nejme1706095 PMID - 28604226
3. Willoughby D, Hewlings S, Kalman D. Body Composition Changes in Weight Loss: Strategies and Supplementation for Maintaining Lean Body Mass, a Brief Review. *Nutrients* 2018;10(12):1876. doi: 10.3390/nu10121876 PMID - 30513859
4. Zhang L, Wang Z, Wang X, et al. Prevalence of Abdominal Obesity in China: Results from a Cross-Sectional Study of Nearly Half a Million Participants. *Obesity* 2019;27(11):1898-905. doi: 10.1002/oby.22620 PMID - 31549787
5. Gouni-Berthold I, Berthold HK. Current Options for the Pharmacotherapy of Obesity. *Curr Pharm Design* 2019;25(18):2019-32. doi: 10.2174/1381612825666190708192630 PMID - 31298150
6. Puhl RM, Heuer CA. Obesity Stigma: Important Considerations for Public Health. *Am J Public Health* 2010;100(6):1019-28. doi: 10.2105/ajph.2009.159491 PMID - 20075322
7. Frieden TR. The Future of Public Health. *New Engl J Medicine* 2015;373(18):1748-54. doi: 10.1056/nejmsa1511248 PMID - 26510022
8. Kirkpatrick CF, Bolick JP, Kris-Etherton PM, et al. Review of current evidence and clinical recommendations on the effects of low-carbohydrate and very-low-carbohydrate (including ketogenic) diets for the management of body weight and other cardiometabolic risk factors: A scientific statement from the National Lipid Association Nutrition and Lifestyle Task Force. *J Clin Lipidol* 2019;13(5):689-711.e1. doi: 10.1016/j.jacl.2019.08.003 PMID - 31611148
9. Ríos-Hoyo A, Gutiérrez-Salmeán G. New Dietary Supplements for Obesity: What We Currently Know. *Curr Obes Reports* 2016;5(2):262-70. doi: 10.1007/s13679-016-0214-y PMID - 27053066
10. Baxter JH, Carlos JL, Thurmond J, et al. Dietary toxicity of calcium β-hydroxy-β-methyl butyrate (CaHMB). *Food Chem Toxicol* 2005;43(12):1731-41. doi: 10.1016/j.fct.2005.05.016 PMID - 16006030
11. Nissen S, Sharp RL, Panton L, et al. β-Hydroxy-β-Methylbutyrate (HMB) Supplementation in Humans Is Safe and May Decrease Cardiovascular Risk Factors. *J Nutrition* 2000;130(8):1937-45. doi: 10.1093/jn/130.8.1937 PMID - 10917905
12. Rathmacher JA, Nissen S, Panton L, et al. Supplementation with a combination of beta-hydroxy-beta-methylbutyrate (HMB), arginine, and glutamine is safe and could improve hematological parameters. *Jpen-parenter Enter* 2004;28(2):65-75. doi: 10.1177/014860710402800265 PMID - 15080599
13. Wu H, Xia Y, Jiang J, et al. Effect of beta-hydroxy-beta-methylbutyrate supplementation on muscle loss in older adults: A systematic review and meta-analysis. *Arch Gerontol Geriat* 2015;61(2):168-75.

For peer review only - http://bmjopen.bmj.com/site/about/guidelines.xhtml

doi: 10.1016/j.archger.2015.06.020 PMID - 26169182

14. Lin Z, Zhao Y, Chen Q. Effects of oral administration of β-hydroxy β-methylbutyrate on lean body mass in older adults: a systematic review and meta-analysis. *Eur Geriatr Med* 2021;12(2):239-51. doi: 10.1007/s41999-020-00409-9 PMID - 33034021
15. Panton LB, Rathmacher JA, Baier S, et al. Nutritional supplementation of the leucine metabolite β-hydroxy-β-methylbutyrate (hmb) during resistance training11Study funded by Metabolic Technologies, Inc. *Nutrition* 2000;16(9):734-39. doi: 10.1016/s0899-9007(00)00376-2 PMID - 10978853
16. Chen C, Lu FC, China DoDCMoHPR. The guidelines for prevention and control of overweight and obesity in Chinese adults. *Biomed Environ Sci Bes* 2004;17 Suppl:1-36.
17. Tremblay MS, Aubert S, Barnes JD, et al. Sedentary Behavior Research Network (SBRN) – Terminology Consensus Project process and outcome. *Int J Behav Nutr Phy* 2017;14(1):75. doi: 10.1186/s12966-017-0525-8 PMID - 28599680
18. Yoshimura E, Kumahara H, Tobina T, et al. Lifestyle Intervention Involving Calorie Restriction with or without Aerobic Exercise Training Improves Liver Fat in Adults with Visceral Adiposity. *J Obes* 2014;2014:1-8. doi: 10.1155/2014/197216 PMID - 24864199
19. China" GAoSo. Chinese National Physical Fitness Evaluation Standard (CNPFES). Beijing2003.
20. Molarius A, Seidell JC, Sans S, et al. Waist and hip circumferences, and waist-hip ratio in 19 populations of the WHO MONICA Project. *Int J Obesity* 1999;23(2):116-25. doi: 10.1038/sj.ijo.0800772 PMID - 10078844
21. Lee PH, Macfarlane DJ, Lam TH, et al. Validity of the international physical activity questionnaire short form (IPAQ-SF): A systematic review. *Int J Behav Nutr Phy* 2011;8(1):115. doi: 10.1186/1479-5868-8-115 PMID - 22018588
22. Bohannon RW. Sit-to-Stand Test for Measuring Performance of Lower Extremity Muscles. *Percept Motor Skill* 1994;80(1):163-66. doi: 10.2466/pms.1995.80.1.163 PMID - 7624188
23. Watanabe D, Nanri H, Sagayama H, et al. Estimation of Energy Intake by a Food Frequency Questionnaire: Calibration and Validation with the Doubly Labeled Water Method in Japanese Older People. *Nutrients* 2019;11(7):1546. doi: 10.3390/nu11071546 PMID - 31323937
24. Hollaender N, Gonzalez-Maffe J, Jehl V. Quantitative assessment of adverse events in clinical trials: Comparison of methods at an interim and the final analysis. *Biometrical J* 2020;62(3):658-69. doi: 10.1002/bimj.201800234 PMID - 31756032
25. Peng LN, Cheng YC, Yu PC, et al. Oral Nutritional Supplement with β-hydroxy-β-methylbutyrate (HMB) Improves Nutrition, Physical Performance and Ameliorates Intramuscular Adiposity in Pre-Frail Older Adults: A Randomized Controlled Trial. *J Nutrition Heal Aging* 2021:1-7. doi: 10.1007/s12603-021-1621-7
26. Chen L-K, Woo J, Assantachai P, et al. Asian Working Group for Sarcopenia: 2019 Consensus Update on Sarcopenia Diagnosis and Treatment. *J Am Med Dir Assoc* 2020(Geriatr Gerontol Int 17 2017) doi: 10.1016/j.jamda.2019.12.012
27. Association WM. World Medical Association Declaration of Helsinki: Ethical Principles for Medical Research Involving Human Subjects. *Jama* 2013;310(20):2191-94. doi: 10.1001/jama.2013.281053 PMID - 24141714
28. Chan A-W, Tetzlaff JM, Gøtzsche PC, et al. SPIRIT 2013 explanation and elaboration: guidance for protocols of clinical trials. *Bmj Br Medical J* 2013;346(jan08 15):e7586. doi: 10.1136/bmj.e7586

For peer review only

For peer review only - http://bmjopen.bmj.com/site/about/guidelines.xhtml

PMID - 23303884

29. Albert FJ, Morente-Sánchez J, Ortega FB, et al. Usefulness of β-hydroxy-β-methylbutyrate (HMB) supplementation in different sports: an update and practical implications. *Nutrición Hosp* 2015;32(1):20-33. doi: 10.3305/nh.2015.32.1.9101 PMID - 26262692
30. Hashempour A, Hooshmand S, Tabesh MR, et al. Effect of 6-week HMB (beta-hydroxy-beta methylbutyrate) supplementation on muscle strength and body composition in sedentary overweight women. *Obes Medicine* 2019;15:100115. doi: 10.1016/j.obmed.2019.100115
31. Rondanelli M, Nichetti M, Peroni G, et al. Effect of a Food for Special Medical Purposes for Muscle Recovery, Consisting of Arginine, Glutamine and Beta-Hydroxy-Beta-Methylbutyrate on Body Composition and Skin Health in Overweight and Obese Class I Sedentary Postmenopausal Women. *Nutrients* 2021;13(3) doi: 10.3390/nu13030975
32. Hurt RT, Ebbert JO, Croghan I, et al. The Comparison of Segmental Multi-frequency BIA and DXA for Estimating Lean Body Mass and Percent Body Fat in an Ambulatory Population. *Jpen-parenter Enter* 2020 doi: 10.1002/jpen.1994 PMID - 32794583

Figure legends

Figure 1. Flow chart of this trial.

For peer review only

For peer review only - http://bmjopen.bmj.com/site/about/guidelines.xhtml

Table 1 Schedule of enrollment, interventions, and assessments

	Enrolment	Allocation	Post-allocation			
	Visit 1	Visit 2	Visit 3	Visit 4	Visit 5	Visit 6
Timepoint	Screening (-4 to 0 wks.)	Baseline (0 wk.)	3 wks. +/- 2 ds	6 wks. +/- 2 ds	9 wks. +/- 2 ds	12 wks. +/- 2 ds
**Enrollment**						
Consent	√					
Inclusion & exclusion	√					
Demographics	√					
Medical history	√					
Body height	√					
Bodyweight	√					
Blood pressure	√					
Heart rate	√					
BIA	√					
Allocation		√				
**Interventions**						
Group A		●	—	—	—	●
Group B		●	—	—	—	●
**Assessments**						
Inclusion & exclusion		√				
Bodyweight		√		√		√
Blood pressure		√		√		√
Heart rate		√		√		√
WC		√		√		√
HC		√		√		√
IPAQ-SF		√	√	√	√	√
FFQ		√	√	√	√	√
BIA		√		√		√
Sit-to-stand test		√		√		√
HGS		√		√		√
Blood tests		√				√
Adverse events			√	√	√	√
Adherence			√	√	√	√

Abbreviations: BIA: bioimpedance analysis; FFQ: Food Frequency Questionnaire; HC: hip circumference; HGS: handgrip strength; IPAQ-SF: International Physical Activity Questionnaire Short Form; WC: waist circumference

For peer review only - http://bmjopen.bmj.com/site/about/guidelines.xhtml

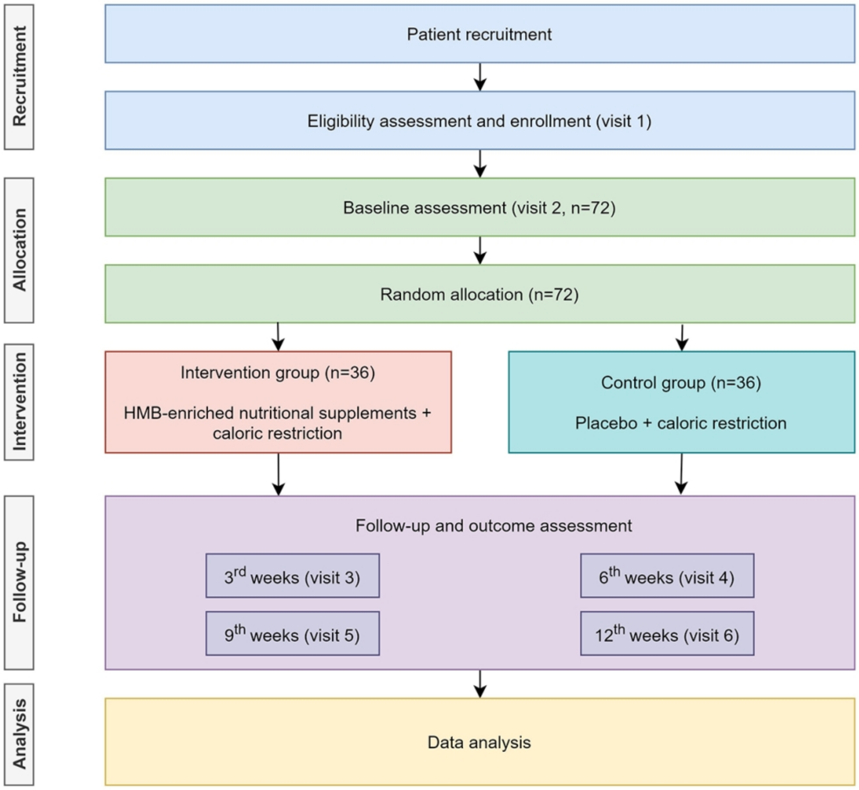

Figure 1. Flow chart of this trial.

Reporting checklist for protocol of a clinical trial.

Based on the SPIRIT guidelines.

Instructions to authors

Complete this checklist by entering the page numbers from your manuscript where readers will find each of the items listed below.

Your article may not currently address all the items on the checklist. Please modify your text to include the missing information. If you are certain that an item does not apply, please write "n/a" and provide a short explanation.

Upload your completed checklist as an extra file when you submit to a journal.

In your methods section, say that you used the SPIRITreporting guidelines, and cite them as:

Chan A-W, Tetzlaff JM, Gøtzsche PC, Altman DG, Mann H, Berlin J, Dickersin K, Hróbjartsson A, Schulz KF, Parulekar WR, Krleža-Jerić K, Laupacis A, Moher D. SPIRIT 2013 Explanation and Elaboration: Guidance for protocols of clinical trials. BMJ. 2013;346:e7586

		Reporting Item	Page Number
**Administrative information**			
Title	#1	Descriptive title identifying the study design, population, interventions, and, if applicable, trial acronym	1
Trial registration	#2a	Trial identifier and registry name. If not yet registered, name of intended registry	2
Trial registration: data set	#2b	All items from the World Health Organization Trial Registration Data Set	N/A
Protocol version	#3	Date and version identifier	16
Funding	#4	Sources and types of financial, material, and other support	17
Roles and responsibilities: contributorship	#5a	Names, affiliations, and roles of protocol contributors	16-17

For peer review only

Roles and responsibilities: sponsor contact information	#5b	Name and contact information for the trial sponsor	1
Roles and responsibilities: sponsor and funder	#5c	Role of study sponsor and funders, if any, in study design; collection, management, analysis, and interpretation of data; writing of the report; and the decision to submit the report for publication, including whether they will have ultimate authority over any of these activities	17
Roles and responsibilities: committees	#5d	Composition, roles, and responsibilities of the coordinating centre, steering committee, endpoint adjudication committee, data management team, and other individuals or groups overseeing the trial, if applicable (see Item 21a for data monitoring committee)	N/A
**Introduction**			
Background and rationale	#6a	Description of research question and justification for undertaking the trial, including summary of relevant studies (published and unpublished) examining benefits and harms for each intervention	3-4
Background and rationale: choice of comparators	#6b	Explanation for choice of comparators	4
Objectives	#7	Specific objectives or hypotheses	4
Trial design	#8	Description of trial design including type of trial (eg, parallel group, crossover, factorial, single group), allocation ratio, and framework (eg, superiority, equivalence, non-inferiority, exploratory)	5
**Methods: Participants, interventions, and outcomes**			
Study setting	#9	Description of study settings (eg, community clinic, academic hospital) and list of countries where data will be collected. Reference to where list of study sites can be obtained	5
Eligibility criteria	#10	Inclusion and exclusion criteria for participants. If applicable, eligibility criteria for study centres and individuals who will	5-7

For peer review only

		perform the interventions (eg, surgeons, psychotherapists)	
Interventions: description	#11a	Interventions for each group with sufficient detail to allow replication, including how and when they will be administered	8
Interventions: modifications	#11b	Criteria for discontinuing or modifying allocated interventions for a given trial participant (eg, drug dose change in response to harms, participant request, or improving / worsening disease)	7
Interventions: adherance	#11c	Strategies to improve adherence to intervention protocols, and any procedures for monitoring adherence (eg, drug tablet return; laboratory tests)	8
Interventions: concomitant care	#11d	Relevant concomitant care and interventions that are permitted or prohibited during the trial	8
Outcomes	#12	Primary, secondary, and other outcomes, including the specific measurement variable (eg, systolic blood pressure), analysis metric (eg, change from baseline, final value, time to event), method of aggregation (eg, median, proportion), and time point for each outcome. Explanation of the clinical relevance of chosen efficacy and harm outcomes is strongly recommended	8-12
Participant timeline	#13	Time schedule of enrolment, interventions (including any run-ins and washouts), assessments, and visits for participants. A schematic diagram is highly recommended (see Figure)	12, Table1
Sample size	#14	Estimated number of participants needed to achieve study objectives and how it was determined, including clinical and statistical assumptions supporting any sample size calculations	12-13
Recruitment	#15	Strategies for achieving adequate participant enrolment to reach target sample size	5
**Methods: Assignment of interventions (for controlled trials)**			
Allocation: sequence generation	#16a	Method of generating the allocation sequence (eg, computer-generated random numbers), and list of any factors for stratification. To reduce predictability of a random sequence, details of any planned restriction (eg, blocking) should be provided in a separate document that is unavailable to those who enrol participants or assign interventions	7

For peer review only

Allocation concealment mechanism	#16b	Mechanism of implementing the allocation sequence (eg, central telephone; sequentially numbered, opaque, sealed envelopes), describing any steps to conceal the sequence until interventions are assigned	7
Allocation: implementation	#16c	Who will generate the allocation sequence, who will enrol participants, and who will assign participants to interventions	7
Blinding (masking)	#17a	Who will be blinded after assignment to interventions (eg, trial participants, care providers, outcome assessors, data analysts), and how	8
Blinding (masking): emergency unblinding	#17b	If blinded, circumstances under which unblinding is permissible, and procedure for revealing a participant's allocated intervention during the trial	8
**Methods: Data collection, management, and analysis**			
Data collection plan	#18a	Plans for assessment and collection of outcome, baseline, and other trial data, including any related processes to promote data quality (eg, duplicate measurements, training of assessors) and a description of study instruments (eg, questionnaires, laboratory tests) along with their reliability and validity, if known. Reference to where data collection forms can be found, if not in the protocol	9
Data collection plan: retention	#18b	Plans to promote participant retention and complete follow-up, including list of any outcome data to be collected for participants who discontinue or deviate from intervention protocols	9
Data management	#19	Plans for data entry, coding, security, and storage, including any related processes to promote data quality (eg, double data entry; range checks for data values). Reference to where details of data management procedures can be found, if not in the protocol	12
Statistics: outcomes	#20a	Statistical methods for analysing primary and secondary outcomes. Reference to where other details of the statistical analysis plan can be found, if not in the protocol	13-14
Statistics: additional analyses	#20b	Methods for any additional analyses (eg, subgroup and adjusted analyses)	14

For peer review only

Statistics: analysis population and missing data	#20c	Definition of analysis population relating to protocol non-adherence (eg, as randomised analysis), and any statistical methods to handle missing data (eg, multiple imputation)	13
**Methods: Monitoring**			
Data monitoring: formal committee	#21a	Composition of data monitoring committee (DMC); summary of its role and reporting structure; statement of whether it is independent from the sponsor and competing interests; and reference to where further details about its charter can be found, if not in the protocol. Alternatively, an explanation of why a DMC is not needed	12
Data monitoring: interim analysis	#21b	Description of any interim analyses and stopping guidelines, including who will have access to these interim results and make the final decision to terminate the trial	12
Harms	#22	Plans for collecting, assessing, reporting, and managing solicited and spontaneously reported adverse events and other unintended effects of trial interventions or trial conduct	11
Auditing	#23	Frequency and procedures for auditing trial conduct, if any, and whether the process will be independent from investigators and the sponsor	N/A
**Ethics and dissemination**			
Research ethics approval	#24	Plans for seeking research ethics committee / institutional review board (REC / IRB) approval	14
Protocol amendments	#25	Plans for communicating important protocol modifications (eg, changes to eligibility criteria, outcomes, analyses) to relevant parties (eg, investigators, REC / IRBs, trial participants, trial registries, journals, regulators)	14
Consent or assent	#26a	Who will obtain informed consent or assent from potential trial participants or authorised surrogates, and how (see Item 32)	14
Consent or assent: ancillary studies	#26b	Additional consent provisions for collection and use of participant data and biological specimens in ancillary studies, if applicable	N/A
Confidentiality	#27	How personal information about potential and enrolled participants will be collected, shared, and maintained in order to protect confidentiality before, during, and after the trial	12

For peer review only

Declaration of interests	#28	Financial and other competing interests for principal investigators for the overall trial and each study site	17
Data access	#29	Statement of who will have access to the final trial dataset, and disclosure of contractual agreements that limit such access for investigators	18
Ancillary and post trial care	#30	Provisions, if any, for ancillary and post-trial care, and for compensation to those who suffer harm from trial participation	N/A
Dissemination policy: trial results	#31a	Plans for investigators and sponsor to communicate trial results to participants, healthcare professionals, the public, and other relevant groups (eg, via publication, reporting in results databases, or other data sharing arrangements), including any publication restrictions	14
Dissemination policy: authorship	#31b	Authorship eligibility guidelines and any intended use of professional writers	N/A
Dissemination policy: reproducible research	#31c	Plans, if any, for granting public access to the full protocol, participant-level dataset, and statistical code	N/A
**Appendices**			
Informed consent materials	#32	Model consent form and other related documentation given to participants and authorised surrogates	N/A
Biological specimens	#33	Plans for collection, laboratory evaluation, and storage of biological specimens for genetic or molecular analysis in the current trial and for future use in ancillary studies, if applicable	N/A

The SPIRIT Explanation and Elaboration paper is distributed under the terms of the Creative Commons Attribution License CC-BY-NC. This checklist was completed on 12. July 2021 using https://www.goodreports.org/, a tool made by the EQUATOR Network in collaboration with Penelope.ai

For peer review only

BMJ Open

β-hydroxy-β-methylbutyrate-enriched nutritional supplements for obese adults during weight loss: study protocol of a randomized, double-blind, placebo-controlled clinical trial

Journal:	*BMJ Open*
Manuscript ID	bmjopen-2021-055420.R1
Article Type:	Protocol
Date Submitted by the Author:	14-Nov-2021
Complete List of Authors:	Jing, Xiaofan; Sichuan University West China Hospital, Department of Clinical Nutrition<br>Liang, Yuxiang; Sichuan University West China Hospital<br>Wang, Renjie; Sichuan University<br>Fu, Hongbo; Sichuan University<br>Jiang, Jiaojiao; Sichuan University<br>Yang, Ming; Sichuan University; Sichuan University
**Primary Subject Heading**:	Nutrition and metabolism
Secondary Subject Heading:	Health services research
Keywords:	Nutritional support < GASTROENTEROLOGY, NUTRITION & DIETETICS, Nutrition < TROPICAL MEDICINE, Nutritional support < ONCOLOGY, Musculoskeletal disorders < ORTHOPAEDIC & TRAUMA SURGERY

SCHOLARONE™
Manuscripts

β-hydroxy-β-methylbutyrate-enriched nutritional supplements for obese adults during weight loss: study protocol of a randomized, double-blind, placebo-controlled clinical trial

Xiaofan Jing [1]; Yuxiang Liang [2]; Renjie Wang [2]; Hongbo Fu [3]; Jiaojiao Jiang [2, *]; Ming Yang [3,4, *]

1. Department of Clinical Nutrition, West China Hospital, Sichuan University, Chengdu, China

2. Rehabilitation Center, West China Hospital, Sichuan University, Chengdu, China

3. National Clinical Research Center for Geriatrics, West China Hospital, Sichuan University, Chengdu, China

4. Center of Gerontology and Geriatrics, West China Hospital, Sichuan University, Chengdu, China

***Corresponding authors:**

Jiaojiao Jiang

Rehabilitation Center, West China Hospital, Sichuan University, No 37 Guoxue Lane, Chengdu 610041, Sichuan, China

Tel: +86 28 8542 2313

Fax: +86 28 8542 2313

Email: jiangjiaojiao1997@163.com

Ming Yang

National Clinical Research Center for Geriatrics, West China Hospital, Sichuan University, No 37 Guoxue Lane, Chengdu 610041, Sichuan, China

Tel: +86 28 8542 2326

Fax: +86 28 8542 2321

Email: yangmier@gmail.com; yangmier@scu.edu.cn

Keywords: sarcopenia; muscle wasting; HMB; obesity; oral nutritional supplements

Word Count: 3909 words

For peer review only

For peer review only - http://bmjopen.bmj.com/site/about/guidelines.xhtml

Abstract

Introduction Obesity is a public health challenge worldwide. To maintain a healthy weight, dieting and lifestyle changes are the first-line interventions; however, these interventions are of poor compliance and may cause body composition changes, mainly skeletal muscle wasting (sarcopenia). Dietary supplements for improving body composition while inducing weight loss are therefore needed. β-hydroxy-β-methylbutyrate (HMB) has been proven to be effective for improving muscle mass and muscle strength in athletes, older adults, and cancer patients. We aim to evaluate the effectiveness and safety of HMB-enriched nutritional supplements for improving muscle mass and muscle function in obese adults during calorie restriction.

Method and analysis A total of 72 Chinese adults with obesity will be randomized to receive HMB-enriched nutritional supplements (65 g/day) or a placebo for 12 weeks. Participants in both groups will also receive caloric restriction based on the individualized nutrition guidance of dieticians. Participants and investigators will be blinded to the allocations. The primary outcome will be the mean change in whole-body skeletal muscle mass (measured by bioelectrical impedance analysis). The secondary outcomes will include the mean change of appendicular skeletal muscle mass, body fat mass, basal metabolic rate, phase angle, muscle function, and serum biomarkers. The enrollment will commence in December 2021 and will proceed until March 2022.

Ethics and dissemination This protocol has been approved by the Biomedical Ethics Committee of West China Hospital (2021-771). All potential subjects will be required to sign a written informed consent. The results of this study will be reported in peer-reviewed academic journals and conferences.

For peer review only - http://bmjopen.bmj.com/site/about/guidelines.xhtml

Trial registration number NCT04953936

Strengths and limitations of this study

- This is the first randomised, double-blind, placebo-controlled trial to explore the possible effectiveness and safety of HMB-enriched nutrition supplements for maintaining skeletal muscle mass and function in obese adults during calorie restriction.
- Using identical doses and appearance of HMB-enriched nutritional supplements and placebos to mask participants and outcome assessors.
- The hydration status of the participants may affect the results of bioimpedance analysis.
- The sample size of this trial is relatively small.

INTRODUCTION

Obesity is a chronic disease featured by abnormal or excess body fat that impairs health. [1] Over the past three decades, obesity has become a common health problem that plagues modern people worldwide [2]. For example, more than one-third (36.5%) of the US adult population is obese defined by a body mass index (BMI) ≥ 30 kg/m^2 [3]. In China, the prevalence of abdominal obesity (defined by a waist circumference ≥ 90 cm and 85 cm for men and women, respectively) among adults is approximately 29%, and the number of obese adults is approximately 277.8 million [4]. Obesity is not only a risk factor for cardiovascular diseases, diabetes, dyslipidemia, chronic kidney disease, and some types of cancer, but also increases the risk of psychological disorders, disability, poor quality of life, and death [1 2 5 6]. Therefore, obesity prevention and control have become one of the key

For peer review only - http://bmjopen.bmj.com/site/about/guidelines.xhtml

contemporary public health challenges [7].

It is well known that increasing physical activity and improving dietary strategies are the keys to the prevention and management of obesity [1]. Many dietary strategies have been proposed for weight loss, such as very low energy diets, ketogenic diets, high protein diets, and high fiber diets. However, individuals who apply these approaches may experience weight loss accompanied by body composition changes, mainly skeletal muscle wasting (sarcopenia) [3]. Sarcopenia may result in a lower resting metabolic rate and a greater tendency to regain body fat. Recently, there is growing evidence that certain nutritional fortifications can improve body composition during weight loss [8]. A variety of over-the-counter dietary supplements for this purpose are available, and they have gained popularity among consumers for weight management [9].

Leucine, a branched-chain essential amino acid, and its active metabolite HMB play an important role in regulating protein synthesis in muscle cells, especially through activation of the mammalian target of rapamycin (mTOR) signal pathway [10]. HMB can also suppress protein degradation pathways by inhibiting intracellular inflammation and caspase-8 activation [11]. However, the rate of conversion of leucine to HMB is very low, and only approximately 5% of leucine in humans is converted to HMB [12]. Studies have found that HMB has the effect of increasing muscle mass and reducing exercise injury [13] [14]. The tolerability and safety of HMB in humans have been well established [15-17]. As early as 1995, the US Food and Drug Administration (FDA) has listed HMB in "Generally Recognized as Safe" (GRAS) ingredients. In 2010, China's former Ministry of Health announced the approval of HMB as a "new resource food", which refers to a newly developed, newly discovered, or newly introduced food without eating habits in Chinese that meets the basic requirements for food while being non-toxic and harmless to humans.

For peer review only - http://bmjopen.bmj.com/site/about/guidelines.xhtml

Studies have shown that HMB supplementation is also beneficial in building muscle strength and may be beneficial in improving body composition. For example, a systematic review that included seven randomized controlled trials (RCTs) showed that HMB supplementation was valuable for maintaining muscle mass in older adults and preventing muscle atrophy due to bed rest or other factors [18]. Another systematic evaluation that included nine RCTs also showed that HMB supplementation improved body composition in older adults [19]. Daily HMB supplementation is supposed to increase the benefits of exercise and to improve body composition in healthy adults in the early stages of regular exercise [20]. Therefore, HMB is expected to be a muscle-protective nutritional fortification that can effectively improve muscle mass and muscle strength in both healthy adults and the elderly. However, evidence regarding the effectiveness of HMB on maintaining skeletal muscle in obese adults during weight loss is limited in the literature. Additionally, very few clinical trials regarding HMB have been conducted in Chinese populations. Therefore, this trial aims to assess the effectiveness and safety of HMB-enriched nutritional supplements for improving muscle mass and muscle function in Chinese adults with obesity during the weight loss process using calorie restriction.

METHODS AND ANALYSIS

Study Design

We designed a single-center, randomized, parallel-group, double-blind, placebo-controlled trial. We plan to recruit 72 Chinese obese adults who intend to perform weight loss via calorie restriction. The participants will be randomly assigned to either the intervention group or the control group at

For peer review only

For peer review only - http://bmjopen.bmj.com/site/about/guidelines.xhtml

a 1:1 ratio and will receive HMB-enriched nutritional supplements or placebo, respectively, for 12 weeks. The flow chart of this trial is presented in **Figure 1** while the overall schedule of this trial is shown in **Table 1**.

Recruitment

This trial will be conducted at West China Hospital, Sichuan University, China. To reach the target sample size, we plan to advertise this trial using the hospital's homepage, WeChat, and flyers inside and outside the hospitals.

Participants

Inclusion Criteria

Individuals who meet all the following criteria will be invited to participate in this study: (1) men and women aged 30-50 years; (2) currently having obesity defined by body mass index (BMI) ≥ 28 kg/m^2 (based on measured body weight and height at booking, using validated scales) [21]; (3) having an intention to lose weight via caloric restriction; (4) healthy and able to walk independently; (5) able to eat independently to meet their energy needs; (6) without significant body weight change in the last 6 month (less than 5% change in body weight); (7) having sedentary habit defined by Sedentary Behavior Research Network [22]; (8) able to collaborate with the research staff. Individuals will be recruited by research nurses or physicians. The study protocol will be provided in advance and individuals who wish to participate will be offered the opportunity to discuss the study and consent to participate. All participants will be required to sign the written informed consent before participation.

For peer review only - http://bmjopen.bmj.com/site/about/guidelines.xhtml

Exclusion Criteria

Individuals with any of the following criteria will be excluded: (1) a history of intolerance to enteral nutrition, food (e.g., lactose intolerance), or being allergic to the components of the HMB-enriched nutritional supplements (e.g., soy or corn); (2) having any implants (e.g., pacemakers); (3) current use of other nutritional supplements or drugs that may have an impact on the effectiveness of the intervention (e.g., glucocorticoids, antineoplastic drugs, antituberculosis drugs, sedatives, antipsychotics, muscle relaxants, monoamine oxidase inhibitors, antibiotics) within 3 months prior to enrollment; (4) secondary obesity caused by diseases (e.g., hypothalamic diseases and hypercortisolism) or drugs (e.g. glucocorticoids, insulin, tricyclic antidepressants, and weight-loss supplements/drugs); (5) clinically visible edema; (6) trauma, surgery, hospitalization, fall, or fracture within 6 months prior to enrollment; (7) pregnancy, having pregnancy plans, or lactation; (8) individuals who are participating in other clinical trials; (9) any acute illness (e.g., acute infection, myocardial infarction, arrhythmia, myocarditis, appendicitis, etc.); (10) swallowing disorders; (11) abnormal thyroid function (including hyperthyroidism and hypothyroidism); (12) history of diabetes, respiratory diseases, cardiovascular diseases, uncontrolled hypertension, kidney diseases, digestive system diseases, renal insufficiency, mental illness, neurological diseases, hematologic diseases, liver diseases (except for fatty liver), chronic infection (e.g., tuberculosis), immune system diseases, joint disorders, any type of tumor; (13) alcohol consumption over two standard alcoholic beverages (20 g of alcohol/day); (14) menopause women; (15) difficulty to comply with the study protocol; and (16) other conditions that indicate the individuals are inappropriate for participation in this study.

For peer review only

For peer review only - http://bmjopen.bmj.com/site/about/guidelines.xhtml

Withdrawal criteria

We will withdraw participants from this trial after randomization for the following reasons: (1) if the participants request to withdraw from this trial; (2) if there is evidence of a complication or the occurrence of an unknown disease, the investigators can decide that the patient cannot continue in the study; (3) if there is an adverse event classified as grade 3 or higher according to the Common Terminology Criteria for Adverse Events (CTCAE) version 5.0; (4) if investigators believe the participants are unsuitable for continuing participating in the study for any other reason.

Randomization and allocation concealment

We will use the stratified blocked randomization method to perform randomization. Random number sequence will be automatically generated by R version 4.0.3 (R Foundation for Statistical Computing, Vienna, Austria) before the inclusion of the first participants. Gender is first used as a stratification factor, and 36 cases of each gender will be included, and then block group randomization will be performed for each gender, with block group lengths set to 2-8 and randomly selected by the computer.

To perform allocation concealment, we will seal the randomization list in sequentially numbered opaque envelopes. The envelopes will be opened sequentially at the beginning of the intervention in the order of entry, and the grouping of participants will be determined according to the allocation scheme inside the envelope.

For peer review only - http://bmjopen.bmj.com/site/about/guidelines.xhtml

Blinding

By using identical doses and appearance of HMB-enriched nutritional supplements and placebos, participants and outcome assessors will be blinded to group allocation.

Interventions

The participants will be randomly assigned into two groups: the intervention group and the control group. The two groups will be randomly labeled A or B. The participants in both groups will perform a caloric restriction diet. They will receive individualized nutritional guidance from professional dietitians by video regarding caloric restriction strategies. The principle for caloric restriction will be based on the equation: energy intake = ideal body weight (i.e., height (cm) -105) × 25 kcal/kg/d [23]. The principle for energy supply ratios of the three major nutrients are as follows: (1) Protein: we recommend an adequate supply of protein (1.2-1.5 g/kg) and using soy protein to partially replace casein; (2) fat: we recommend a 20%-30% energy supply ratio for fat. Also, we recommend increasing foods rich in omega-3 fatty acids; (3) carbohydrate: we recommend determining the supply of carbohydrates based on the intake of protein and fat. The appropriate ratio of carbohydrates for energy supply should be 40%-55% [24]. The source of carbohydrates should be mainly starch-based complex carbohydrates, and the intake of dietary fiber is recommended as 25-30 g/d [24]. Moreover, we recommend strictly limiting the intake of simple sugars (i.e., monosaccharides, disaccharides) in foods or beverages [24].

The compliance will be followed via a WeChat group and telephone. The intervention duration in both groups will be 12 weeks.

For peer review only - http://bmjopen.bmj.com/site/about/guidelines.xhtml

Intervention group

The participants in the intervention group will receive oral HMB-enriched nutritional supplements (65 g, once daily, 239 kcal), which include soybean isolate, protein, whey protein, casein, HMB, L-glutamine, wheat oligopeptide, medium-chain fatty acids, vitamin D, vitamin E, konjac powder, and fish oil powder. The nutritional supplements contain 24 g protein, 9.3 g fat, and 14.6 g carbohydrates, with energy ratios of 40.2%, 35.0%, and 24.8%, respectively. Among them, n-3 polyunsaturated fatty acids accounted for 43% in fat, and dietary fiber accounted for 9.5g in total carbohydrate.

Control group

The participants in the control group will receive a placebo (maltodextrin 65 g once daily, 250 kcal) in the same opaque plastic bottle as the intervention.

Outcomes

As shown in Table 1, the assessors, who will be blinded to the group allocation, will ask the participants to complete questionnaires (including demographic characteristics, physical activity, and energy intake), anthropometric measurement (including body weight, height, waist circumference, and hip circumference), heart rate, blood pressure, handgrip strength measurement, sit-to-stand test, and body composition at the baseline, the 6-week, and the end of the 12-week intervention. Additionally, participants will complete self-reported questionnaires at the 3-week and the 9-week.

Moreover, fasting blood will be taken by a nurse at baseline and the end of 12-week intervention after intervention, for laboratory analysis of biomarkers of glucose metabolism (fasting glucose and

For peer review only - http://bmjopen.bmj.com/site/about/guidelines.xhtml

insulin), albumin, hemoglobin, alanine aminotransferase (ALT), aspartate aminotransferase (AST), gamma-glutamyl transpeptidase (GGT), Cystatin C, creatinine, estimated glomerular filtration rate (eGFR), lipid profiles, and full blood counts. The fasting blood will be sent to the clinical laboratory of West China Hospital, Sichuan University within 2 h after withdrawal. These biomarkers will be tested using standard methods. Moreover, Homeostasis Model Assessment of Insulin Resistance (HOMA-IR) will be calculated using the equation: fasting glucose (mg/dL) × fasting insulin (μIU/mL)/405.

Primary outcome

The mean change in whole-body skeletal muscle mass (SMM) from the beginning to the end of the 12-week intervention will be measured for all participants. The whole-body lean body mass will be measured by trained research staff using a bioimpedance analysis device (BIA; InBody 770; Biospace Co., Seoul, Korea). The participants will be guided through the BIA test. They will be asked to stand upright at least 5 m before testing, then to step on the footplate barefoot with their heels on the rear sole electrodes and their hand holding the hand electrode. Their arms should be straight and should not touch the side of the body during the test.

Secondary outcomes

We will measure the following secondary outcomes:

- The mean change in appendicular skeletal muscle mass (ASMM), trunk skeletal muscle mass (TSMM), total body fat mass (BFM), appendicular body fat mass (ABFM), trunk body fat mass (TBFM), basal metabolic rate (BMR), phase angle (PA), and body weight (BW) from the

For peer review only

For peer review only - http://bmjopen.bmj.com/site/about/guidelines.xhtml

beginning to weeks 6 and 12, as well as the mean change in whole-body SMM from the beginning to week 6, will be measured for all participants. These data will be measured using InBody 770 (Biospace Co., Seoul, Korea).

- The mean change in handgrip strength (HGS) from the beginning to weeks 6 and 12 will be measured for all participants. A trained nurse will measure HGS using a digital handheld dynamometer (EH101; Xiangshan Inc, Guangdong, China) to the nearest 0.1 kg according to the recommendation of the Chinese National Physical Fitness Evaluation Standard [25]. The participants will be asked to perform three measurements for each hand, with rest periods of ≥ 30 s between each measurement. The HGS will be determined as the maximum value of the six records.
- The mean change in waist circumference and hip circumference from the beginning to weeks 6 and 12 will be measured for all participants. A trained nurse will measure waist circumference using a tape at the level midway between the lower rib margin and the iliac crest with the participant breathing out gently. Hip circumference will be measured as the maximum circumference over the buttocks [26].
- The mean change in physical activity from the beginning to weeks 3, 6, 9, and 12 will be measured for all participants using the self-reported International Physical Activity Questionnaire Short Form (IPAQ-SF) [27].
- The mean change in the score of the sit-to-stand test from the beginning to weeks 6 and 12 will be measured for all participants. A trained nurse will perform the 30-second sit-to-stand test [28], which is a classic test for measuring the performance of lower extremity muscles.
- The mean change in energy intake from the beginning to weeks 3, 6, 9, and 12 will be measured

For peer review only - http://bmjopen.bmj.com/site/about/guidelines.xhtml

for all participants using the self-reported Food Frequency Questionnaire (FFQ) [29].

- The mean change of lipid profiles, fasting glucose, fasting insulin, HOMA-IR, and albumin from the beginning to the end of the 12-week intervention will be measured for all participants.

Safety outcomes

We will record all adverse events during the entire study period. The participants will be asked to complete a self-reported questionnaire regarding any adverse event every 3 weeks. The participants will also be encouraged to report adverse events to the research staff via telephone when there is any adverse event occurrence. We will rank the severity of adverse events according to the Common Terminology Criteria for Adverse Events (CTCAE) version 5.0. The cause of the adverse events will be ranked as unknown, definitely not related, probably not related, possibly related, probably related, and definitely related [30].

Moreover, we will report the mean change in the laboratory tests regarding adverse events from the beginning to the end of the 12-week intervention: hemoglobin, AST, ALT, GGT, Cystatin C, creatinine, and eGFR.

Feasibility outcomes

We will record the recruitment rate and completion rate to assess the feasibility of this trial. Additionally, the adherence rate to the interventions will be calculated. Moreover, we will record the reasons for participants' ineligibility and for dropping out of this trial.

For peer review only - http://bmjopen.bmj.com/site/about/guidelines.xhtml

Data Collection, Management, and Monitoring

Research coordinators will formally obtain the participants' informed consent before collecting and recording data in the case report form (CRF). The assessment of outcomes will be done by assessors who are unaware of the assignment of the groups.

To ensure that participants adhere to the research strategy, all participants will be included in the WeChat group and the investigators will call them at least one day beforehand for each appointment and follow-up session. Participants who do not attend follow-up sessions will be asked for their reasons and telephone contacts will be made to encourage adherence to the intervention and attendance at appointments. We will also contact participants via WeChat or telephone once a week to determine if any adverse events have occurred.

For the study's quality, monitoring and auditing of the data will be carried out. Prior to the beginning of the study, there will be an introductory meeting. At every important point in the trial, such as participant enrolment, the midway point, and the conclusion, monitoring staff will visit the institution. In addition, monitoring staff will ensure consistency in the documentation of data contained in the CRF and source document, as well as ensuring that the entire study process is conducted according to the protocol.

Sample Size Estimation

To date, no studies have been done to determine whether HMB-enriched nutritional supplements are effective and safe for improving muscle mass and muscle function in obese adults during weight loss. To determine the sample size, we examined similar studies on HMB and referred to the study by Peng et al. that evaluated the effectiveness of HMB for improving skeletal muscle mass and

For peer review only - http://bmjopen.bmj.com/site/about/guidelines.xhtml

quality in pre-frail older adults [12]. Using a 0.05 significance level, 80% statistical power, and 25% attrition rate, a sample size of 70 has been assumed using an assumed effective size of 0.93. Moreover, to achieve a gender-balanced distribution within and between groups, a random stratification method including 36 samples per gender is proposed, adding up to 72 samples in total.

Statistical Analysis Plan

The statistical analysis will be performed by an independent statistical expert blinded to the group allocation. SPSS version 26.0 (IBM Corp, Armonk, NY) and R version 4.0.3 (R Foundation for Statistical Computing, Vienna, Austria) will be used for statistical analysis.

Each group will be summarized in terms of demographic characteristics and baseline variables. For comparisons of continuous variables, two-sample T-tests or Mann–Whitney U tests will be used, whereas for categorical variables, the χ^2 test or Fisher's exact test will be used.

As aforementioned, the primary outcome will be in SMM from the beginning to the end of the 12-week intervention. The independent two-sample T-test or Mann-Whitney U-test will be used based on the distributional normality. We will make adjustments based on the covariates if there are significant demographic differences between the groups or if baseline variables differ significantly. In the primary analysis, the full analysis set (FAS) will be the main data set. Participants initially assigned to either group will be included in the FAS. However, our analysis will not use the data of ineligible participants (i.e., those who are wrongly enrolled because of measurement or human error), subjects who received less than 75% of the planned intervention, or subjects without the primary outcome measurement. For sensitivity analysis, we will use a protocol-specific set to compare with the results from the FAS. A per-protocol analysis will include participants who achieve the

For peer review only - http://bmjopen.bmj.com/site/about/guidelines.xhtml

completion of the study without major deviations from protocol, who provide the primary outcome, and who receive at least 75% of the planned intervention. A significance level of two-sided $p < 0.05$ will be used. If needed, the multiple imputation method will be adopted for missing data.

We will use the same methods for analyzing secondary outcomes as we do for analyzing the primary outcome. A paired t-test or Wilcoxon signed-rank tests will be used to assess the changes in outcome measures within groups before and after the intervention. To validate trend differences per visit, we will use repeated measures analysis of variance with post hoc tests.

Moreover, subgroup analysis will be done to identify whether gender or Asian Working Group on Sarcopenia (AWGS)-defined sarcopenia [31] at baseline affect the clinical response to HMB-enriched nutritional supplements.

ETHICS AND DISSEMINATION

This trial has been registered in ClinicalTrials.gov (reference NCT04953936). This trial will be conducted following the Declaration of Helsinki [32]. This study protocol was drafted according to Standard Protocol Items: Recommendations for Interventional Trials (SPIRIT) statement [33]. It has been approved by the Biomedical Ethics Committee of West China Hospital (reference 2021-771). Any protocol amendments will be reapproved by the Biomedical Ethics Committee of West China Hospital and will be reflected in the participants' informed consent form. We will obtain signed consent forms from all participants regarding the modification. The English version of the informed consent form is presented in **Supplementary Material**.

We intend to publish the results of this trial in appropriate academic peer-reviewed journals and conferences.

For peer review only - http://bmjopen.bmj.com/site/about/guidelines.xhtml

Patient and Public Involvement

We did not involve patients or the public in the planning and design of this study.

DISCUSSION

This study will be a randomized, double-blind, parallel-group, placebo-controlled trial aiming to explore the effectiveness and safety of HMB-enriched nutritional supplements for improving muscle mass and muscle strength in obese adults during the weight loss process using calorie restriction. It will determine the feasibility of future large-scale clinical trials on HMB-based interventions for improving body composition (especially muscle health) in obese adults when performing weight loss plans.

The effectiveness of HMB for improving muscle mass and muscle strength has been well studied in athletes, healthy adults, older adults, and cancer patients [34]. However, we found only two clinical trials addressing the effectiveness of HMB on muscle health in obese adults [34 35]. In 2019, an RCT conducted in Iran demonstrated that HMB supplements for six weeks reduced weight, waist, and abdominal circumference, and even increased strength without resistance training, without affecting lean mass, in sedentary and overweight women [35]. Most recently, Rondanelli et al. [36] conducted an RCT in obese or overweight Italian menopausal women. Using oral nutrition supplements containing arginine, glutamine, and HMB for four weeks, they observed that visceral adipose tissue and BFM were statistically significantly reduced, whereas fat-free mass (mainly muscle mass) remained the same.

Our trial has some strengths. First, it will be a pioneer to evaluate the effectiveness of HMB-enriched

For peer review only - http://bmjopen.bmj.com/site/about/guidelines.xhtml

nutritional supplements for improving muscle health in Chinese obese adults. Second, we will use a double-blind design to minimize bias due to demand characteristics, the placebo effect, and measurement bias. Last, we will focus on not only muscle mass and fat mass but also muscle function (i.e., handgrip strength and physical performance).

This trial also has some limitations. First, this will be a single-center trial and the sample size is relatively small. Therefore, the representativeness of our study sample may be limited. Second, participants in this study may be heterogeneous, given the wide age range. This heterogeneity should be considered when interpreting the results. Third, it might not be possible to completely control the dietary and physical activity of all participants. However, we will record the physical activity and energy intakes and will adjust for them as potential confounders. Fourth, the muscle mass and fat mass will be measured with a BIA device rather than a dual-energy X-ray absorptiometry (DXA) scanner. The hydration status of the participants may affect the results of bioimpedance analysis. However, a recent study showed that DXA and segmental multifrequency BIA (InBody 770, which will be applied in this trial) were equally accurate for estimating muscle mass and fat mass [37].

Trial status

This protocol is version 1.2, written on November 9, 2021. The enrollment will commence in December 2021 and will proceed until March 2022.

Author contributions

Conceptualization, M.Y.; methodology, M.Y., J.J., and X.J.; validation, M.Y., and J.J.; formal analysis, J.J., and X.J.; investigation, X.J., H.F., R.W., and Y.L.; resources, M.Y.; data curation,

For peer review only - http://bmjopen.bmj.com/site/about/guidelines.xhtml

J.J.; writing-original draft preparation, X.J., and M.Y.; writing-review and editing, J.J.; visualization, X.J.; supervision, M.Y.; project administration, J.J.; funding acquisition, M.Y. All authors have read and agreed to the published version of the manuscript.

Funding

This trial was supported by the National Key R&D Program of China (Grant number: 2018YFC2002104) and the K&D Program of the Sichuan Science and Technology Department (Grant number: 2020YFS0573). The sponsor had no role in the design of this trial and will not take part in the methods, data collection, and data analysis of this trial.

Competing interests

The authors declare no conflict of interest.

Acknowledgements

None.

Patients consent for publication

Not required.

Data availability statement

The data of this trial will be available from the corresponding author on reasonable request.

For peer review only - http://bmjopen.bmj.com/site/about/guidelines.xhtml

Ethics approval

This study protocol has been approved by the Biomedical Ethics Committee of West China Hospital (reference 2021-771). This study protocol has been registered in ClinicalTrials.gov (reference NCT04953936).

Open access

This is an open access article distributed in accordance with the Creative Commons Attribution Non-Commercial (CC BY-NC 4.0) license, which permits others to distribute, remix, adapt, build upon this work non-commercially, and license their derivative works on different terms, provided the original work is properly cited, appropriate credit is given, any changes made indicated, and the use is non-commercial. See: http://creativecommons.org/licenses/by-nc/4.0/.

ORCID ID

Ming Yang https://orcid.org/0000-0002-1875-1692

References

1. Wharton S, Lau DCW, Vallis M, et al. Obesity in adults: a clinical practice guideline. *Cmaj* 2020;192(31):E875-E91. doi: 10.1503/cmaj.191707 PMID - 32753461
2. Gregg EW, Shaw JE. Global Health Effects of Overweight and Obesity. *New Engl J Medicine* 2017;377(1):80-81. doi: 10.1056/nejme1706095 PMID - 28604226
3. Willoughby D, Hewlings S, Kalman D. Body Composition Changes in Weight Loss: Strategies and Supplementation for Maintaining Lean Body Mass, a Brief Review. *Nutrients* 2018;10(12):1876. doi: 10.3390/nu10121876 PMID - 30513859
4. Zhang L, Wang Z, Wang X, et al. Prevalence of Abdominal Obesity in China: Results from a Cross-Sectional Study of Nearly Half a Million Participants. *Obesity* 2019;27(11):1898-905. doi: 10.1002/oby.22620 PMID - 31549787
5. Gouni-Berthold I, Berthold HK. Current Options for the Pharmacotherapy of Obesity. *Curr Pharm*

For peer review only - http://bmjopen.bmj.com/site/about/guidelines.xhtml

Design 2019;25(18):2019-32. doi: 10.2174/1381612825666190708192630 PMID - 31298150
6. Puhl RM, Heuer CA. Obesity Stigma: Important Considerations for Public Health. *Am J Public Health* 2010;100(6):1019-28. doi: 10.2105/ajph.2009.159491 PMID - 20075322
7. Frieden TR. The Future of Public Health. *New Engl J Medicine* 2015;373(18):1748-54. doi: 10.1056/nejmsa1511248 PMID - 26510022
8. Kirkpatrick CF, Bolick JP, Kris-Etherton PM, et al. Review of current evidence and clinical recommendations on the effects of low-carbohydrate and very-low-carbohydrate (including ketogenic) diets for the management of body weight and other cardiometabolic risk factors: A scientific statement from the National Lipid Association Nutrition and Lifestyle Task Force. *J Clin Lipidol* 2019;13(5):689-711.e1. doi: 10.1016/j.jacl.2019.08.003 PMID - 31611148
9. Ríos-Hoyo A, Gutiérrez-Salmeán G. New Dietary Supplements for Obesity: What We Currently Know. *Curr Obes Reports* 2016;5(2):262-70. doi: 10.1007/s13679-016-0214-y PMID - 27053066
10. He X, Duan Y, Yao K, et al. β-Hydroxy-β-methylbutyrate, mitochondrial biogenesis, and skeletal muscle health. *Amino Acids* 2016;48(3):653-64. doi: 10.1007/s00726-015-2126-7 PMID - 26573541
11. Landi F, Calvani R, Picca A, et al. Beta-hydroxy-beta-methylbutyrate and sarcopenia. *Curr Opin Clin Nutr* 2019;22(1):37-43. doi: 10.1097/mco.0000000000000524 PMID - 30489401
12. Peng LN, Cheng YC, Yu PC, et al. Oral Nutritional Supplement with β-hydroxy-β-methylbutyrate (HMB) Improves Nutrition, Physical Performance and Ameliorates Intramuscular Adiposity in Pre-Frail Older Adults: A Randomized Controlled Trial. *J Nutrition Heal Aging* 2021:1-7. doi: 10.1007/s12603-021-1621-7
13. Holeček M. Beta-hydroxy-beta-methylbutyrate supplementation and skeletal muscle in healthy and muscle - wasting conditions. *J Cachexia Sarcopenia Muscle* 2017;8(4):529-41. doi: 10.1002/jcsm.12208 PMID - 28493406
14. Cavallucci V, Pani G. The Leucine Catabolite and Dietary Supplement β-Hydroxy-β-Methyl Butyrate (HMB) as an Epigenetic Regulator in Muscle Progenitor Cells. *Metabolites* 2021;11(8):512. doi: 10.3390/metabo11080512 PMID - 34436453
15. Baxter JH, Carlos JL, Thurmond J, et al. Dietary toxicity of calcium β-hydroxy-β-methyl butyrate (CaHMB). *Food Chem Toxicol* 2005;43(12):1731-41. doi: 10.1016/j.fct.2005.05.016 PMID - 16006030
16. Nissen S, Sharp RL, Panton L, et al. β-Hydroxy-β-Methylbutyrate (HMB) Supplementation in Humans Is Safe and May Decrease Cardiovascular Risk Factors. *J Nutrition* 2000;130(8):1937-45. doi: 10.1093/jn/130.8.1937 PMID - 10917905
17. Rathmacher JA, Nissen S, Panton L, et al. Supplementation with a combination of beta-hydroxy-beta-methylbutyrate (HMB), arginine, and glutamine is safe and could improve hematological parameters. *Jpen-parenter Enter* 2004;28(2):65-75. doi: 10.1177/014860710402800265 PMID - 15080599
18. Wu H, Xia Y, Jiang J, et al. Effect of beta-hydroxy-beta-methylbutyrate supplementation on muscle loss in older adults: A systematic review and meta-analysis. *Arch Gerontol Geriat* 2015;61(2):168-75. doi: 10.1016/j.archger.2015.06.020 PMID - 26169182
19. Lin Z, Zhao Y, Chen Q. Effects of oral administration of β-hydroxy β-methylbutyrate on lean body mass in older adults: a systematic review and meta-analysis. *Eur Geriatr Med* 2021;12(2):239-51. doi: 10.1007/s41999-020-00409-9 PMID - 33034021

For peer review only - http://bmjopen.bmj.com/site/about/guidelines.xhtml

20. Panton LB, Rathmacher JA, Baier S, et al. Nutritional supplementation of the leucine metabolite β-hydroxy-β-methylbutyrate (hmb) during resistance training11Study funded by Metabolic Technologies, Inc. *Nutrition* 2000;16(9):734-39. doi: 10.1016/s0899-9007(00)00376-2 PMID - 10978853

21. Chen C, Lu FC, China DoDCMoHPR. The guidelines for prevention and control of overweight and obesity in Chinese adults. *Biomed Environ Sci Bes* 2004;17 Suppl:1-36.

22. Tremblay MS, Aubert S, Barnes JD, et al. Sedentary Behavior Research Network (SBRN) – Terminology Consensus Project process and outcome. *Int J Behav Nutr Phy* 2017;14(1):75. doi: 10.1186/s12966-017-0525-8 PMID - 28599680

23. Yoshimura E, Kumahara H, Tobina T, et al. Lifestyle Intervention Involving Calorie Restriction with or without Aerobic Exercise Training Improves Liver Fat in Adults with Visceral Adiposity. *J Obes* 2014;2014:1-8. doi: 10.1155/2014/197216 PMID – 24864199

24. Chinese Society of Health Management, Clinical Nutrition Branch of Chinese Nutrition Society, Medical Nutrition Industry Branch of the National Association of Health Industry and Enterprise Management,et al. Expert consensus on the procedure of body weight management among patients with overweight or obesity. Chinese Journal of Health Management, 2021; 15(4):317-322.

25. General Administration of Sport of China. Chinese National Physical Fitness Evaluation Standard (CNPFES). Beijing 2003.

26. Molarius A, Seidell JC, Sans S, et al. Waist and hip circumferences, and waist-hip ratio in 19 populations of the WHO MONICA Project. *Int J Obesity* 1999;23(2):116-25. doi: 10.1038/sj.ijo.0800772 PMID - 10078844

27. Lee PH, Macfarlane DJ, Lam TH, et al. Validity of the international physical activity questionnaire short form (IPAQ-SF): A systematic review. *Int J Behav Nutr Phy* 2011;8(1):115. doi: 10.1186/1479-5868-8-115 PMID - 22018588

28. Bohannon RW. Sit-to-Stand Test for Measuring Performance of Lower Extremity Muscles. *Percept Motor Skill* 1994;80(1):163-66. doi: 10.2466/pms.1995.80.1.163 PMID - 7624188

29. Watanabe D, Nanri H, Sagayama H, et al. Estimation of Energy Intake by a Food Frequency Questionnaire: Calibration and Validation with the Doubly Labeled Water Method in Japanese Older People. *Nutrients* 2019;11(7):1546. doi: 10.3390/nu11071546 PMID - 31323937

30. Hollaender N, Gonzalez-Maffe J, Jehl V. Quantitative assessment of adverse events in clinical trials: Comparison of methods at an interim and the final analysis. *Biometrical J* 2020;62(3):658-69. doi: 10.1002/bimj.201800234 PMID - 31756032

31. Chen L-K, Woo J, Assantachai P, et al. Asian Working Group for Sarcopenia: 2019 Consensus Update on Sarcopenia Diagnosis and Treatment. *J Am Med Dir Assoc* 2020(Geriatr Gerontol Int 17 2017) doi: 10.1016/j.jamda.2019.12.012

32. Association WM. World Medical Association Declaration of Helsinki: Ethical Principles for Medical Research Involving Human Subjects. *Jama* 2013;310(20):2191-94. doi: 10.1001/jama.2013.281053 PMID - 24141714

33. Chan A-W, Tetzlaff JM, Gøtzsche PC, et al. SPIRIT 2013 explanation and elaboration: guidance for protocols of clinical trials. *Bmj Br Medical J* 2013;346(jan08 15):e7586. doi: 10.1136/bmj.e7586 PMID - 23303884

34. Albert FJ, Morente-Sánchez J, Ortega FB, et al. Usefulness of β-hydroxy-β-methylbutyrate (HMB)

For peer review only - http://bmjopen.bmj.com/site/about/guidelines.xhtml

supplementation in different sports: an update and practical implications. *Nutrición Hosp* 2015;32(1):20-33. doi: 10.3305/nh.2015.32.1.9101 PMID - 26262692

35. Hashempour A, Hooshmand S, Tabesh MR, et al. Effect of 6-week HMB (beta-hydroxy-beta methylbutyrate) supplementation on muscle strength and body composition in sedentary overweight women. *Obes Medicine* 2019;15:100115. doi: 10.1016/j.obmed.2019.100115

36. Rondanelli M, Nichetti M, Peroni G, et al. Effect of a Food for Special Medical Purposes for Muscle Recovery, Consisting of Arginine, Glutamine and Beta-Hydroxy-Beta-Methylbutyrate on Body Composition and Skin Health in Overweight and Obese Class I Sedentary Postmenopausal Women. *Nutrients* 2021;13(3) doi: 10.3390/nu13030975

37. Hurt RT, Ebbert JO, Croghan I, et al. The Comparison of Segmental Multi-frequency BIA and DXA for Estimating Lean Body Mass and Percent Body Fat in an Ambulatory Population. *Jpen-parenter Enter* 2020 doi: 10.1002/jpen.1994 PMID - 32794583

Figure legends

Figure 1. Flow chart of this trial.

For peer review only

For peer review only - http://bmjopen.bmj.com/site/about/guidelines.xhtml

Table 1 Schedule of enrollment, interventions, and assessments

	Enrolment	Allocation	Post-allocation			
	Visit 1	Visit 2	Visit 3	Visit 4	Visit 5	Visit 6
Timepoint	Screening (-4 to 0 wks.)	Baseline (0 wk.)	3 wks. +/- 2 ds	6 wks. +/- 2 ds	9 wks. +/- 2 ds	12 wks. +/- 2 ds
**Enrollment**						
Consent	√					
Inclusion & exclusion	√					
Demographics	√					
Medical history	√					
Body height	√					
Bodyweight	√					
Blood pressure	√					
Heart rate	√					
BIA	√					
Allocation		√				
**Interventions†**						
HMB nutritional supplement + caloric restriction		●	—	—	—	●
Placebo + caloric restriction		●	—	—	—	●
**Assessments**						
Inclusion & exclusion		√				
Bodyweight		√		√		√
Blood pressure		√		√		√
Heart rate		√		√		√
WC		√		√		√
HC		√		√		√
IPAQ-SF		√	√	√	√	√
FFQ		√	√	√	√	√
BIA		√		√		√
Sit-to-stand test		√		√		√
HGS		√		√		√
Blood tests‡		√				√
Adverse events*			√	√	√	√
Adherence*			√	√	√	√

Abbreviations: BIA: bioimpedance analysis; FFQ: Food Frequency Questionnaire; HC: hip circumference; HGS: handgrip strength; IPAQ-SF: International Physical Activity Questionnaire Short Form; WC: waist circumference

† Both intervention groups will also receive dietary instruction.

‡ The blood tests will include glucose metabolism (fasting glucose and insulin), albumin, hemoglobin, alanine aminotransferase, aspartate aminotransferase, gamma-glutamyl transpeptidase, Cystatin C, creatinine, estimated glomerular filtration rate, lipid profiles, and full blood counts.

* Weekly WeChat or telephone contacts will be performed to assess adverse events and adherence (including dietary compliance).

For peer review only - http://bmjopen.bmj.com/site/about/guidelines.xhtml

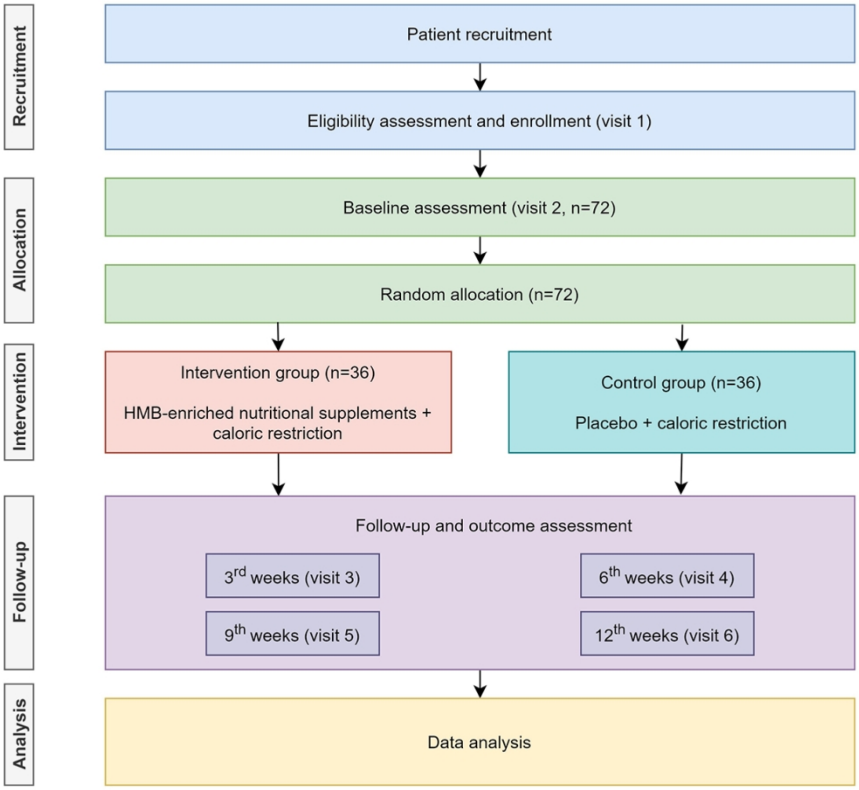

Figure 1. Flow chart of this trial.

Reporting checklist for protocol of a clinical trial.

Based on the SPIRIT guidelines.

Instructions to authors

Complete this checklist by entering the page numbers from your manuscript where readers will find each of the items listed below.

Your article may not currently address all the items on the checklist. Please modify your text to include the missing information. If you are certain that an item does not apply, please write "n/a" and provide a short explanation.

Upload your completed checklist as an extra file when you submit to a journal.

In your methods section, say that you used the SPIRITreporting guidelines, and cite them as:

Chan A-W, Tetzlaff JM, Gøtzsche PC, Altman DG, Mann H, Berlin J, Dickersin K, Hróbjartsson A, Schulz KF, Parulekar WR, Krleža-Jerić K, Laupacis A, Moher D. SPIRIT 2013 Explanation and Elaboration: Guidance for protocols of clinical trials. BMJ. 2013;346:e7586

		Reporting Item	Page Number
**Administrative information**			
Title	#1	Descriptive title identifying the study design, population, interventions, and, if applicable, trial acronym	1

For peer review only

Trial registration	#2a	Trial identifier and registry name. If not yet registered, name of intended registry	2
Trial registration: data set	#2b	All items from the World Health Organization Trial Registration Data Set	N/A
Protocol version	#3	Date and version identifier	16
Funding	#4	Sources and types of financial, material, and other support	17
Roles and responsibilities: contributorship	#5a	Names, affiliations, and roles of protocol contributors	16-17
Roles and responsibilities: sponsor contact information	#5b	Name and contact information for the trial sponsor	1
Roles and responsibilities: sponsor and funder	#5c	Role of study sponsor and funders, if any, in study design; collection, management, analysis, and interpretation of data; writing of the report; and the decision to submit the report for publication, including whether they will have ultimate authority over any of these activities	17
Roles and responsibilities: committees	#5d	Composition, roles, and responsibilities of the coordinating centre, steering committee, endpoint adjudication committee, data management team, and	N/A

For peer review only

		other individuals or groups overseeing the trial, if applicable (see Item 21a for data monitoring committee)	
**Introduction**			
Background and rationale	#6a	Description of research question and justification for undertaking the trial, including summary of relevant studies (published and unpublished) examining benefits and harms for each intervention	3-4
Background and rationale: choice of comparators	#6b	Explanation for choice of comparators	4
Objectives	#7	Specific objectives or hypotheses	4
Trial design	#8	Description of trial design including type of trial (eg, parallel group, crossover, factorial, single group), allocation ratio, and framework (eg, superiority, equivalence, non-inferiority, exploratory)	5
**Methods: Participants, interventions, and outcomes**			
Study setting	#9	Description of study settings (eg, community clinic, academic hospital) and list of countries where data will be collected. Reference to where list of study sites can be obtained	5

For peer review only

Eligibility criteria	#10	Inclusion and exclusion criteria for participants. If applicable, eligibility criteria for study centres and individuals who will perform the interventions (eg, surgeons, psychotherapists)	5-7
Interventions: description	#11a	Interventions for each group with sufficient detail to allow replication, including how and when they will be administered	8
Interventions: modifications	#11b	Criteria for discontinuing or modifying allocated interventions for a given trial participant (eg, drug dose change in response to harms, participant request, or improving / worsening disease)	7
Interventions: adherance	#11c	Strategies to improve adherence to intervention protocols, and any procedures for monitoring adherence (eg, drug tablet return; laboratory tests)	8
Interventions: concomitant care	#11d	Relevant concomitant care and interventions that are permitted or prohibited during the trial	8
Outcomes	#12	Primary, secondary, and other outcomes, including the specific measurement variable (eg, systolic blood pressure), analysis metric (eg, change from baseline, final value, time to event), method of aggregation (eg, median, proportion), and time point for each outcome. Explanation of the clinical relevance of chosen efficacy and harm outcomes is strongly recommended	8-12

For peer review only

Participant timeline	#13	Time schedule of enrolment, interventions (including any run-ins and washouts), assessments, and visits for participants. A schematic diagram is highly recommended (see Figure)	12 , Table1
Sample size	#14	Estimated number of participants needed to achieve study objectives and how it was determined, including clinical and statistical assumptions supporting any sample size calculations	12-13
Recruitment	#15	Strategies for achieving adequate participant enrolment to reach target sample size	5
**Methods: Assignment of interventions (for controlled trials)**			
Allocation: sequence generation	#16a	Method of generating the allocation sequence (eg, computer-generated random numbers), and list of any factors for stratification. To reduce predictability of a random sequence, details of any planned restriction (eg, blocking) should be provided in a separate document that is unavailable to those who enrol participants or assign interventions	7
Allocation concealment mechanism	#16b	Mechanism of implementing the allocation sequence (eg, central telephone; sequentially numbered, opaque, sealed envelopes), describing any steps to conceal the sequence until interventions are assigned	7

For peer review only

Allocation: implementation	#16c	Who will generate the allocation sequence, who will enrol participants, and who will assign participants to interventions	7
Blinding (masking)	#17a	Who will be blinded after assignment to interventions (eg, trial participants, care providers, outcome assessors, data analysts), and how	8
Blinding (masking): emergency unblinding	#17b	If blinded, circumstances under which unblinding is permissible, and procedure for revealing a participant's allocated intervention during the trial	8
**Methods: Data collection, management, and analysis**			
Data collection plan	#18a	Plans for assessment and collection of outcome, baseline, and other trial data, including any related processes to promote data quality (eg, duplicate measurements, training of assessors) and a description of study instruments (eg, questionnaires, laboratory tests) along with their reliability and validity, if known. Reference to where data collection forms can be found, if not in the protocol	9
Data collection plan: retention	#18b	Plans to promote participant retention and complete follow-up, including list of any outcome data to be	9

For peer review only

		collected for participants who discontinue or deviate from intervention protocols	
Data management	#19	Plans for data entry, coding, security, and storage, including any related processes to promote data quality (eg, double data entry; range checks for data values). Reference to where details of data management procedures can be found, if not in the protocol	12
Statistics: outcomes	#20a	Statistical methods for analysing primary and secondary outcomes. Reference to where other details of the statistical analysis plan can be found, if not in the protocol	13-14
Statistics: additional analyses	#20b	Methods for any additional analyses (eg, subgroup and adjusted analyses)	14
Statistics: analysis population and missing data	#20c	Definition of analysis population relating to protocol non-adherence (eg, as randomised analysis), and any statistical methods to handle missing data (eg, multiple imputation)	13
**Methods: Monitoring**			
Data monitoring: formal committee	#21a	Composition of data monitoring committee (DMC); summary of its role and reporting structure; statement of whether it is independent from the sponsor and competing interests; and reference to where further details about its charter can be found, if not in the	12

For peer review only

		protocol. Alternatively, an explanation of why a DMC is not needed	
Data monitoring: interim analysis	#21b	Description of any interim analyses and stopping guidelines, including who will have access to these interim results and make the final decision to terminate the trial	12
Harms	#22	Plans for collecting, assessing, reporting, and managing solicited and spontaneously reported adverse events and other unintended effects of trial interventions or trial conduct	11
Auditing	#23	Frequency and procedures for auditing trial conduct, if any, and whether the process will be independent from investigators and the sponsor	N/A
**Ethics and dissemination**			
Research ethics approval	#24	Plans for seeking research ethics committee / institutional review board (REC / IRB) approval	14
Protocol amendments	#25	Plans for communicating important protocol modifications (eg, changes to eligibility criteria, outcomes, analyses) to relevant parties (eg, investigators, REC / IRBs, trial participants, trial registries, journals, regulators)	14

For peer review only

Consent or assent	#26a	Who will obtain informed consent or assent from potential trial participants or authorised surrogates, and how (see Item 32)	14
Consent or assent: ancillary studies	#26b	Additional consent provisions for collection and use of participant data and biological specimens in ancillary studies, if applicable	N/A
Confidentiality	#27	How personal information about potential and enrolled participants will be collected, shared, and maintained in order to protect confidentiality before, during, and after the trial	12
Declaration of interests	#28	Financial and other competing interests for principal investigators for the overall trial and each study site	17
Data access	#29	Statement of who will have access to the final trial dataset, and disclosure of contractual agreements that limit such access for investigators	18
Ancillary and post trial care	#30	Provisions, if any, for ancillary and post-trial care, and for compensation to those who suffer harm from trial participation	N/A
Dissemination policy: trial results	#31a	Plans for investigators and sponsor to communicate trial results to participants, healthcare professionals, the public, and other relevant groups (eg, via publication, reporting in results databases, or other data sharing arrangements), including any publication restrictions	14

For peer review only

Dissemination policy: authorship	#31b	Authorship eligibility guidelines and any intended use of professional writers	N/A
Dissemination policy: reproducible research	#31c	Plans, if any, for granting public access to the full protocol, participant-level dataset, and statistical code	N/A

Appendices

Informed consent materials	#32	Model consent form and other related documentation given to participants and authorised surrogates	Supplementary file
Biological specimens	#33	Plans for collection, laboratory evaluation, and storage of biological specimens for genetic or molecular analysis in the current trial and for future use in ancillary studies, if applicable	N/A

The SPIRIT Explanation and Elaboration paper is distributed under the terms of the Creative Commons Attribution License CC-BY-NC. This checklist was completed on 12. July 2021 using https://www.goodreports.org/, a tool made by the EQUATOR Network in collaboration with Penelope.ai

For peer review only

BMJ Open

β-hydroxy-β-methylbutyrate-enriched nutritional supplements for obese adults during weight loss: study protocol of a randomized, double-blind, placebo-controlled clinical trial

Journal:	*BMJ Open*
Manuscript ID	bmjopen-2021-055420.R2
Article Type:	Protocol
Date Submitted by the Author:	30-May-2022
Complete List of Authors:	Jing, Xiaofan; Sichuan University West China Hospital, Department of Clinical Nutrition<br>Liang, Yuxiang; Sichuan University West China Hospital<br>Wang, Renjie; Sichuan University<br>Fu, Hongbo; Sichuan University<br>Jiang, Jiaojiao; Sichuan University<br>Yang, Ming; Sichuan University; Sichuan University
**Primary Subject Heading**:	Nutrition and metabolism
Secondary Subject Heading:	Health services research
Keywords:	Nutritional support < GASTROENTEROLOGY, NUTRITION & DIETETICS, Nutrition < TROPICAL MEDICINE, Nutritional support < ONCOLOGY, Musculoskeletal disorders < ORTHOPAEDIC & TRAUMA SURGERY

SCHOLARONE™
Manuscripts

β-hydroxy-β-methylbutyrate-enriched nutritional supplements for obese adults during weight loss: study protocol of a randomized, double-blind, placebo-controlled clinical trial

Xiaofan Jing [1]; Yuxiang Liang [2]; Renjie Wang [2]; Hongbo Fu [3]; Jiaojiao Jiang [2, *]; Ming Yang [3,4, *]

1. Department of Clinical Nutrition, West China Hospital, Sichuan University, Chengdu, China

2. Rehabilitation Center, West China Hospital, Sichuan University, Chengdu, China

3. National Clinical Research Center for Geriatrics, West China Hospital, Sichuan University, Chengdu, China

4. Center of Gerontology and Geriatrics, West China Hospital, Sichuan University, Chengdu, China

***Corresponding authors:**

Jiaojiao Jiang

Rehabilitation Center, West China Hospital, Sichuan University, No 37 Guoxue Lane, Chengdu 610041, Sichuan, China

Tel: +86 28 8542 2313

Fax: +86 28 8542 2313

Email: jiangjiaojiao1997@163.com

Ming Yang

National Clinical Research Center for Geriatrics, West China Hospital, Sichuan University, No 37 Guoxue Lane, Chengdu 610041, Sichuan, China

Tel: +86 28 8542 2326

Fax: +86 28 8542 2321

Email: yangmier@gmail.com; yangmier@scu.edu.cn

Keywords: sarcopenia; muscle wasting; HMB; obesity; oral nutritional supplements

Word Count: 3586 words

For peer review only

For peer review only - http://bmjopen.bmj.com/site/about/guidelines.xhtml

Abstract

Introduction Obesity is a public health challenge worldwide. To maintain a healthy weight, dieting and lifestyle changes are the first-line interventions; however, these interventions are of poor compliance and may cause body composition changes, mainly skeletal muscle wasting (sarcopenia). Dietary supplements for improving body composition while inducing weight loss are therefore needed. β-hydroxy-β-methylbutyrate (HMB) has been proven to be effective for improving muscle mass and muscle strength in athletes, older adults, and cancer patients. We aim to evaluate the effectiveness and safety of HMB-enriched nutritional supplements for improving muscle mass and muscle function in obese adults during calorie restriction.

Method and analysis A total of 72 Chinese adults with obesity will be randomized to receive HMB-enriched nutritional supplements (65 g/day) or a placebo for 12 weeks. Participants in both groups will also receive caloric restrictions based on the individualized nutrition guidance of dieticians. Participants and investigators will be blinded to the allocations. The primary outcome will be the mean change in whole-body skeletal muscle mass (measured by bioelectrical impedance analysis). The secondary outcomes will include the mean change of appendicular skeletal muscle mass, body fat mass, basal metabolic rate, phase angle, muscle function, and serum biomarkers. The enrollment will commence in December 2021 and will proceed until March 2022.

Ethics and dissemination This protocol has been approved by the Biomedical Ethics Committee of West China Hospital (2021-771). All potential subjects will be required to sign a written informed consent. The results of this study will be reported in peer-reviewed academic journals and conferences.

For peer review only - http://bmjopen.bmj.com/site/about/guidelines.xhtml

Trial registration number NCT04953936

Strengths and limitations of this study

- This is the first randomized, double-blind, placebo-controlled trial to explore the possible effectiveness and safety of HMB-enriched nutrition supplements for maintaining skeletal muscle mass and function in obese adults during calorie restriction.
- Using identical doses and appearance of HMB-enriched nutritional supplements and placebos to mask participants and outcome assessors.
- The hydration status of the participants may affect the results of the bioimpedance analysis.
- The sample size of this trial is relatively small.

INTRODUCTION

Obesity is a chronic disease featured by abnormal or excess body fat that impairs health. [1] Over the past three decades, obesity has become a common health problem that plagues modern people worldwide [2]. For example, more than one-third (36.5%) of the US adult population is obese defined by a body mass index (BMI) $\geq$ 30 kg/m^2 [3]. In China, the prevalence of abdominal obesity (defined by a waist circumference $\geq$ 90 cm and 85 cm for men and women, respectively) among adults is approximately 29%, and the number of adults with abdominal obesity (using the same definition) is approximately 277.8 million [4]. Obesity is not only a risk factor for cardiovascular diseases, diabetes, dyslipidemia, chronic kidney disease, and some types of cancer, but also increases the risk of psychological disorders, disability, poor quality of life, and death [1 2 5 6]. Therefore, obesity

For peer review only - http://bmjopen.bmj.com/site/about/guidelines.xhtml

prevention and control have become one of the key contemporary public health challenges [7].

It is well known that increasing physical activity and improving dietary strategies are the keys to the prevention and management of obesity [1]. Many dietary strategies have been proposed for weight loss, such as very low energy diets, ketogenic diets, high protein diets, and high fiber diets. However, individuals who apply these approaches may experience weight loss accompanied by body composition changes, mainly skeletal muscle wasting (sarcopenia) [3]. Sarcopenia may result in a lower resting metabolic rate and a greater tendency to regain body fat. Recently, there is growing evidence that certain nutritional fortifications can improve body composition during weight loss [8]. A variety of over-the-counter dietary supplements for this purpose are available, and they have gained popularity among consumers for weight management [9].

Leucine, a branched-chain essential amino acid, and its active metabolite HMB play an important role in regulating protein synthesis in muscle cells, especially through activation of the mammalian target of rapamycin (mTOR) signal pathway [10]. HMB can also suppress protein degradation pathways by inhibiting intracellular inflammation and caspase-8 activation [11]. However, the rate of conversion of leucine to HMB is very low, and only approximately 5% of leucine in humans is converted to HMB [12]. Studies have found that HMB has the effect of increasing muscle mass and reducing exercise injury [13] [14]. The tolerability and safety of HMB in humans have been well established [15-17]. As early as 1995, the US Food and Drug Administration (FDA) has listed HMB in “Generally Recognized as Safe” (GRAS) ingredients. In 2010, China’s former Ministry of Health announced the approval of HMB as a “new resource food”, which refers to a newly developed, newly discovered, or newly introduced food that is not traditionally consumed in China and that meets the basic requirements for food while being non-toxic and harmless to humans.

For peer review only - http://bmjopen.bmj.com/site/about/guidelines.xhtml

Studies have shown that HMB supplementation is also beneficial in building muscle strength and may be beneficial in improving body composition. For example, a systematic review that included seven randomized controlled trials (RCTs) showed that HMB supplementation was valuable for maintaining muscle mass in older adults and preventing muscle atrophy due to bed rest or other factors [18]. Another systematic evaluation that included nine RCTs also showed that HMB supplementation improved body composition in older adults [19]. Daily HMB supplementation is supposed to increase the benefits of exercise and improve body composition in healthy adults in the early stages of regular exercise [20]. Therefore, HMB is expected to be a muscle-protective nutritional fortification that can effectively improve muscle mass and muscle strength in both healthy adults and the elderly. However, evidence regarding the effectiveness of HMB on maintaining skeletal muscle in obese adults during weight loss is limited in the literature. Additionally, very few clinical trials regarding HMB have been conducted in Chinese populations. Therefore, this trial aims to assess the effectiveness and safety of HMB-enriched nutritional supplements for improving muscle mass and muscle function in Chinese adults with obesity during the weight loss process using calorie restriction.

METHODS AND ANALYSIS

Study Design

We designed a single-center, randomized, parallel-group, double-blind, placebo-controlled trial. We plan to recruit 72 Chinese obese adults who intend to perform weight loss via calorie restriction. The participants will be randomly assigned to either the intervention group or the control group at

For peer review only - http://bmjopen.bmj.com/site/about/guidelines.xhtml

a 1:1 ratio and will receive HMB-enriched nutritional supplements or placebo, respectively, for 12 weeks. The flow chart of this trial is presented in **Figure 1** while the overall schedule of this trial is shown in **Table 1**.

Recruitment

This trial will be conducted at West China Hospital, Sichuan University, China. To reach the target sample size, we plan to advertise this trial using the hospital's homepage, WeChat, and flyers inside and outside the hospitals.

Participants

Inclusion Criteria

Individuals who meet all the following criteria will be invited to participate in this study: (1) men and women aged 30-50 years; (2) currently having obesity defined by body mass index (BMI) ≥ 28 kg/m^2 (based on measured body weight and height at booking, using validated scales) [21]; (3) having an intention to lose weight via caloric restriction; (4) healthy and able to walk independently; (5) able to eat independently to meet their energy needs; (6) without significant body weight change in the last 6 month (less than 5% change in body weight); (7) having sedentary habit defined by Sedentary Behavior Research Network [22]; (8) able to collaborate with the research staff. Individuals will be recruited by research nurses or physicians. The study protocol will be provided in advance and individuals who wish to participate will be offered the opportunity to discuss the study and consent to participate. All participants will be required to sign the written informed consent before participation.

For peer review only

For peer review only - http://bmjopen.bmj.com/site/about/guidelines.xhtml

Exclusion Criteria

Individuals with any of the following criteria will be excluded: (1) a history of intolerance to enteral nutrition, food (e.g., lactose intolerance), or being allergic to the components of the HMB-enriched nutritional supplements (e.g., soy or corn); (2) having any implants (e.g., pacemakers, implantable cardioverter defibrillators, and dental implants); (3) current use of other nutritional supplements or drugs that may have an impact on the effectiveness of the intervention (e.g., glucocorticoids, antineoplastic drugs, antituberculosis drugs, sedatives, antipsychotics, muscle relaxants, monoamine oxidase inhibitors, antibiotics) within 3 months prior to enrollment; (4) secondary obesity caused by diseases (e.g., hypothalamic diseases and hypercortisolism) or drugs (e.g. glucocorticoids, insulin, tricyclic antidepressants, and weight-loss supplements/drugs); (5) clinically visible edema; (6) trauma, surgery, hospitalization, fall, or fracture within 6 months prior to enrollment; (7) pregnancy, having pregnancy plans, or lactation; (8) individuals who are participating in other clinical trials; (9) any acute illness (e.g., acute infection, myocardial infarction, arrhythmia, myocarditis, appendicitis, etc.); (10) swallowing disorders; (11) abnormal thyroid function (including hyperthyroidism and hypothyroidism); (12) history of diabetes, respiratory diseases, cardiovascular diseases, uncontrolled hypertension, kidney diseases, digestive system diseases, renal insufficiency, mental illness, neurological diseases, hematologic diseases, liver diseases (except for fatty liver), chronic infection (e.g., tuberculosis), immune system diseases, joint disorders, any type of tumor; (13) alcohol consumption over two standard alcoholic beverages (20 g of alcohol/day); (14) menopause women; (15) difficulty to comply with the study protocol; and (16) other conditions that indicate the individuals are inappropriate for participation in this study.

For peer review only

For peer review only - http://bmjopen.bmj.com/site/about/guidelines.xhtml

Withdrawal criteria

We will withdraw participants from this trial after randomization for the following reasons: (1) if the participants request to withdraw from this trial; (2) if there is evidence of a complication or the occurrence of an unknown disease, the investigators can decide that the patient cannot continue in the study; (3) if there is an adverse event classified as grade 3 or higher according to the Common Terminology Criteria for Adverse Events (CTCAE) version 5.0; (4) if investigators believe the participants are unsuitable for continuing participating in the study for any other reason.

Randomization and allocation concealment

We will use the stratified blocked randomization method to perform randomization. A random number sequence will be automatically generated by R version 4.0.3 (R Foundation for Statistical Computing, Vienna, Austria) before the inclusion of the first participants. Gender is first used as a stratification factor, and 36 cases of each gender will be included, and then block group randomization will be performed for each gender, with block group lengths set to 2-8 and randomly selected by the computer.

To perform allocation concealment, we will seal the randomization list in sequentially numbered opaque envelopes. The envelopes will be opened sequentially at the beginning of the intervention in the order of entry, and the grouping of participants will be determined according to the allocation scheme inside the envelope.

For peer review only - http://bmjopen.bmj.com/site/about/guidelines.xhtml

Blinding

By using identical doses and appearance of HMB-enriched nutritional supplements and placebos, participants and outcome assessors will be blind to group allocation.

Interventions

The participants will be randomly assigned into two groups: the intervention group and the control group. The two groups will be randomly labeled A or B. The participants in both groups will perform a caloric restriction diet. They will receive individualized nutritional guidance from professional dietitians by video regarding caloric restriction strategies. The principle for caloric restriction will be based on the equation: energy intake = ideal body weight (i.e., height (cm） -105) × 25 kcal/kg/d [23]. The compliance will be followed via a WeChat group and telephone. The intervention duration in both groups will be 12 weeks.

Intervention group

The participants in the intervention group will receive oral HMB-enriched nutritional supplements (65 g, once daily) in powder form packaging in opaque plastic bottles, which include soybean isolate, protein, whey protein, casein, HMB, L-glutamine, wheat oligopeptide, medium-chain fatty acids, vitamin D, vitamin E, konjac powder, and fish oil powder.

Control group

The participants in the control group will receive a placebo (maltodextrin 65 g once daily) in powder form packaging in the same opaque plastic bottles as the intervention.

For peer review only - http://bmjopen.bmj.com/site/about/guidelines.xhtml

Outcomes

As shown in Table 1, the assessors, who will be blinded to the group allocation, will ask the participants to complete questionnaires (including demographic characteristics, physical activity, and energy intake), anthropometric measurements (including body weight, height, waist circumference, and hip circumference), heart rate, blood pressure, handgrip strength measurement, sit-to-stand test, and body composition at the baseline, the 6-week, and the end of the 12-week intervention. Additionally, participants will complete self-reported questionnaires at the 3-week and the 9-week.

Moreover, fasting blood will be taken by a nurse at baseline and at the end of the 12-week intervention after intervention, for laboratory analysis of biomarkers of glucose metabolism (fasting glucose and insulin), albumin, hemoglobin, alanine aminotransferase (ALT), aspartate aminotransferase (AST), gamma-glutamyl transpeptidase (GGT), Cystatin C, creatinine, estimated glomerular filtration rate (eGFR), lipid profiles, and full blood counts. The fasting blood will be sent to the clinical laboratory of West China Hospital, Sichuan University within 2 h after withdrawal. These biomarkers will be tested using standard methods. Moreover, the Homeostasis Model Assessment of Insulin Resistance (HOMA-IR) will be calculated using the equation: fasting glucose (mg/dL) × fasting insulin (μIU/mL)/405.

Primary outcome

The mean change in whole-body skeletal muscle mass (SMM) from the beginning to the end of the 12-week intervention will be measured for all participants. The whole-body lean body mass will be measured by trained research staff using a bioimpedance analysis device (BIA; InBody 770;

For peer review only - http://bmjopen.bmj.com/site/about/guidelines.xhtml

Biospace Co., Seoul, Korea). The participants will be guided through the BIA test. They will be asked to stand upright at least 5 min before testing, then to step on the footplate barefoot with their heels on the rear sole electrodes and their hand holding the hand electrode. Their arms should be straight and should not touch the side of the body during the test.

Secondary outcomes

We will measure the following secondary outcomes:

- The mean change in appendicular skeletal muscle mass (ASMM), trunk skeletal muscle mass (TSMM), total body fat mass (BFM), appendicular body fat mass (ABFM), trunk body fat mass (TBFM), basal metabolic rate (BMR), phase angle (PA), and body weight (BW) from the beginning to weeks 6 and 12, as well as the mean change in whole-body SMM from the beginning to week 6, will be measured for all participants. These data will be measured using InBody 770 (Biospace Co., Seoul, Korea).
- The mean change in handgrip strength (HGS) from the beginning to weeks 6 and 12 will be measured for all participants. A trained nurse will measure HGS using a digital handheld dynamometer (EH101; Xiangshan Inc, Guangdong, China) to the nearest 0.1 kg according to the recommendation of the Chinese National Physical Fitness Evaluation Standard [24]. The participants will be asked to perform three measurements for each hand, with rest periods of ≥ 30 s between each measurement. The HGS will be determined as the maximum value of the six records.
- The mean change in waist circumference and hip circumference from the beginning to weeks 6 and 12 will be measured for all participants. A trained nurse will measure waist circumference

For peer review only - http://bmjopen.bmj.com/site/about/guidelines.xhtml

using a tape at the level midway between the lower rib margin and the iliac crest with the participant breathing out gently. Hip circumference will be measured as the maximum circumference over the buttocks [25].

- The mean change in physical activity from the beginning to weeks 3, 6, 9, and 12 will be measured for all participants using the self-reported International Physical Activity Questionnaire Short Form (IPAQ-SF) [26].
- The mean change in the score of the sit-to-stand test from the beginning to weeks 6 and 12 will be measured for all participants. A trained nurse will perform the 30-second sit-to-stand test [27], which is a classic test for measuring the performance of lower extremity muscles.
- The mean change in energy intake from the beginning to weeks 3, 6, 9, and 12will be measured for all participants using the self-reported Food Frequency Questionnaire (FFQ) [28].
- The mean change of lipid profiles, fasting glucose, fasting insulin, HOMA-IR, and albumin from the beginning to the end of the 12-week intervention will be measured for all participants.

Safety outcomes

We will record all adverse events during the entire study period. The participants will be asked to complete a self-reported questionnaire regarding any adverse event every 3 weeks. The participants will also be encouraged to report adverse events to the research staff via telephone when there is any adverse event occurrence. We will rank the severity of adverse events according to the Common Terminology Criteria for Adverse Events (CTCAE) version 5.0. The cause of the adverse events will be ranked as unknown, definitely not related, probably not related, possibly related, probably related, definitely related [29].

For peer review only - http://bmjopen.bmj.com/site/about/guidelines.xhtml
For peer review only

Moreover, we will report the mean change in the laboratory tests regarding adverse events from the beginning to the end of the 12-week intervention: hemoglobin, AST, ALT, GGT, Cystatin C, creatinine, and eGFR.

Feasibility outcomes

We will record the recruitment rate and completion rate to assess the feasibility of this trial. Additionally, the adherence rate to the interventions will be calculated. Moreover, we will record the reasons for participants' ineligibility and for dropping out of this trial.

Data Collection, Management, and Monitoring

Research coordinators will formally obtain the participants' informed consent before collecting and recording data in the case report form (CRF). The assessment of outcomes will be done by assessors who are unaware of the assignment of the groups.

To ensure that participants adhere to the research strategy, all participants will be included in the WeChat group and the investigators will call them at least one day beforehand for each appointment and follow-up session. Participants who do not attend follow-up sessions will be asked for their reasons and telephone contacts will be made to encourage adherence to the intervention and attendance at appointments. We will also contact participants via WeChat or telephone once a week to determine if any AEs have occurred and to assess their compliance by asking each participant to complete a daily compliance record and the IPAQ-SF and FFQ questionnaires.

For the study's quality, monitoring and auditing of the data will be carried out. Prior to the beginning of the study, there will be an introductory meeting. At every important point in the trial, such as

For peer review only

participant enrolment, the midway point, and the conclusion, monitoring staff will visit the institution. In addition, monitoring staff will ensure consistency in the documentation of data contained in the CRF and source document, as well as ensuring that the entire study process is conducted according to the protocol.

Sample Size Estimation

To date, no studies have been done to determine whether HMB-enriched nutritional supplements are effective and safe for improving muscle mass and muscle function in obese adults during weight loss. To determine the sample size, we examined similar studies on HMB and referred to the study by Peng et al. that evaluated the effectiveness of HMB for improving skeletal muscle mass and quality in pre-frail older adults [12]. Using a 0.05 significance level, 80% statistical power, and 25% attrition rate, a sample size of 70 has been assumed using an assumed effective size of 0.93. Moreover, to achieve a gender-balanced distribution within and between groups, a random stratification method including 36 samples per gender is proposed, adding up to 72 samples in total.

Statistical Analysis Plan

The statistical analysis will be performed by an independent statistical expert blinded to the group allocation. SPSS version 26.0 (IBM Corp, Armonk, NY) and R version 4.0.3 (R Foundation for Statistical Computing, Vienna, Austria) will be used for statistical analysis.

Each group will be summarized in terms of demographic characteristics and baseline variables. For comparisons of continuous variables, two-sample T-tests or Mann–Whitney U tests will be used, whereas for categorical variables, the χ^2 test or Fisher's exact test will be used.

For peer review only - http://bmjopen.bmj.com/site/about/guidelines.xhtml

As aforementioned, the primary outcome will be in SMM from the beginning to the end of the 12-week intervention. The independent two-sample T-test or Mann-Whitney U-test will be used based on the distributional normality. We will make adjustments based on the covariates if there are significant demographic differences between the groups or if baseline variables differ significantly. In the primary analysis, the full analysis set (FAS) will be the main data set. Participants initially assigned to either group will be included in the FAS. However, our analysis will not use the data of ineligible participants (i.e., those who are wrongly enrolled because of measurement or human error), subjects who received less than 75% of the planned intervention, or subjects without the primary outcome measurement. For sensitivity analysis, we will use a protocol-specific set to compare with the results from the FAS. A per-protocol analysis will include participants who achieved the completion of the study without major deviations from protocol, who provided the primary outcome, and who received at least 75% of the planned intervention. The rate of protocol compliance in each group will be calculated. A significance level of two-sided $p < 0.05$ will be used. If needed, the multiple imputation method will be adopted for missing data.

We will use the same methods for analyzing secondary outcomes as we did for analyzing the primary outcome. A paired t-test or Wilcoxon signed-rank test will be used to assess the changes in outcome measures within groups before and after the intervention. To validate trend differences per visit, we will use repeated measures analysis of variance with post hoc tests.

Moreover, subgroup analysis will be done to identify whether gender or Asian Working Group on Sarcopenia (AWGS)-defined sarcopenia [30] at baseline would affect the clinical response to HMB-enriched nutritional supplements.

For peer review only - http://bmjopen.bmj.com/site/about/guidelines.xhtml

ETHICS AND DISSEMINATION

This trial has been registered on ClinicalTrials.gov (reference NCT04953936). This trial will be conducted following the Declaration of Helsinki [31]. This study protocol was drafted according to Standard Protocol Items: Recommendations for Interventional Trials (SPIRIT) statement [32]. It has been approved by the Biomedical Ethics Committee of West China Hospital (reference 2021-771). Any protocol amendments will be reapproved by the Biomedical Ethics Committee of West China Hospital and will be reflected in the participants' informed consent form. We will obtain signed consent forms from all participants regarding the modification.

We intend to publish the results of this trial in appropriate academic peer-reviewed journals and conferences.

Patient and Public Involvement

We did not involve patients or the public in the planning and design of this study.

DISCUSSION

This study will be a randomized, double-blind, parallel-group, placebo-controlled trial aiming to explore the effectiveness and safety of HMB-enriched nutritional supplements for improving muscle mass and muscle strength in obese adults during the weight loss process using calorie restriction. It will determine the feasibility of future large-scale clinical trials on HMB-based interventions for improving body composition (especially muscle health) in obese adults when performing weight loss plans.

The effectiveness of HMB for improving muscle mass and muscle strength has been well studied in

For peer review only

For peer review only - http://bmjopen.bmj.com/site/about/guidelines.xhtml

athletes, healthy adults, older adults, and cancer patients [33]. However, we found only two clinical trials addressing the effectiveness of HMB on muscle health in obese adults [34 35]. In 2019, an RCT conducted in Iran demonstrated that supplementing with HMB for six weeks reduced weight, waist, and abdominal circumference, and even increased strength without resistance training, and without affecting lean mass, in sedentary and overweight women [34]. Most recently, Rondanelli et al. [35] conducted an RCT in obese or overweight Italian menopausal women. Using an oral nutrition supplement containing arginine, glutamine, and HMB for four weeks, they observed that visceral adipose tissue and BFM were statistically significantly reduced, whereas fat-free mass (mainly muscle mass) remained the same.

Our trial has some strengths. First, it will be the first to evaluate the effectiveness of HMB-enriched nutritional supplements for improving muscle health in Chinese obese adults. Second, we will use a double-blinded design to minimize bias due to demand characteristics, the placebo effect, and measurement bias. Last, we will focus on not only muscle mass and fat mass but also muscle function (i.e., handgrip strength and physical performance).

This trial also has some limitations. First, this will be a single-center trial and the sample size is relatively small. Therefore, the representativeness of our study sample may be limited. Second, participants in this study may be heterogeneous, given the wide age range. This heterogeneity should be considered when interpreting the results. Third, it might not be possible to completely control the dietary and physical activity of all participants. However, we will record the physical activity and energy intakes and will adjust for them as potential confounders. Fourth, the muscle mass and fat mass will be measured with a BIA device rather than a dual-energy X-ray absorptiometry (DXA) scanner. The hydration status of the participants may affect the results of the bioimpedance analysis.

For peer review only - http://bmjopen.bmj.com/site/about/guidelines.xhtml

However, a recent study showed that DXA and segmental multifrequency BIA (InBody 770, which will be applied in this trial) were equally accurate for estimating muscle mass and fat mass [36].

Trial status

This protocol is version 1.2, written on November 9, 2021. The enrollment will commence in December 2021 and will proceed until March 2022.

Author contributions

Conceptualization, M.Y.; methodology, M.Y., J.J., and X.J.; validation, M.Y., and J.J.; formal analysis, J.J., and X.J.; investigation, X.J., H.F., R.W., and Y.L.; resources, M.Y.; data curation, J.J.; writing-original draft preparation, X.J., and M.Y.; writing-review and editing, J.J.; visualization, X.J.; supervision, M.Y.; project administration, J.J.; funding acquisition, M.Y. All authors have read and agreed to the published version of the manuscript.

Funding

This trial was supported by the National Key R&D Program of China (Grant number: 2018YFC2002104) and the K&D Program of the Sichuan Science and Technology Department (Grant number: 2020YFS0573). The sponsor had no role in the design of this trial and will not take part in the methods, data collection, and data analysis of this trial.

Competing interests

The authors declare no conflict of interest.

For peer review only - http://bmjopen.bmj.com/site/about/guidelines.xhtml

Acknowledgments

None.

Patients consent for publication

Not required.

Data availability statement

The data of this trial will be available from the corresponding author on reasonable request.

Ethics approval

This study protocol has been approved by the Biomedical Ethics Committee of West China Hospital (reference 2021-771). This study protocol has been registered on ClinicalTrials.gov (reference NCT04953936).

Open access

This is an open-access article distributed in accordance with the Creative Commons Attribution Non-Commercial (CC BY-NC 4.0) license, which permits others to distribute, remix, adapt, build upon this work non-commercially, and license their derivative works on different terms, provided the original work is properly cited, appropriate credit is given, any changes made indicated, and the use is non-commercial. See http://creativecommons.org/licenses/by-nc/4.0/.

For peer review only - http://bmjopen.bmj.com/site/about/guidelines.xhtml

ORCID ID

Ming Yang https://orcid.org/0000-0002-1875-1692

References

1. Wharton S, Lau DCW, Vallis M, et al. Obesity in adults: a clinical practice guideline. *Cmaj* 2020;192(31):E875-E91. doi: 10.1503/cmaj.191707 PMID - 32753461

2. Gregg EW, Shaw JE. Global Health Effects of Overweight and Obesity. *New Engl J Medicine* 2017;377(1):80-81. doi: 10.1056/nejme1706095 PMID - 28604226

3. Willoughby D, Hewlings S, Kalman D. Body Composition Changes in Weight Loss: Strategies and Supplementation for Maintaining Lean Body Mass, a Brief Review. *Nutrients* 2018;10(12):1876. doi: 10.3390/nu10121876 PMID - 30513859

4. Zhang L, Wang Z, Wang X, et al. Prevalence of Abdominal Obesity in China: Results from a Cross-Sectional Study of Nearly Half a Million Participants. *Obesity* 2019;27(11):1898-905. doi: 10.1002/oby.22620 PMID - 31549787

5. Gouni-Berthold I, Berthold HK. Current Options for the Pharmacotherapy of Obesity. *Curr Pharm Design* 2019;25(18):2019-32. doi: 10.2174/1381612825666190708192630 PMID - 31298150

6. Puhl RM, Heuer CA. Obesity Stigma: Important Considerations for Public Health. *Am J Public Health* 2010;100(6):1019-28. doi: 10.2105/ajph.2009.159491 PMID - 20075322

7. Frieden TR. The Future of Public Health. *New Engl J Medicine* 2015;373(18):1748-54. doi: 10.1056/nejmsa1511248 PMID - 26510022

8. Kirkpatrick CF, Bolick JP, Kris-Etherton PM, et al. Review of current evidence and clinical recommendations on the effects of low-carbohydrate and very-low-carbohydrate (including ketogenic) diets for the management of body weight and other cardiometabolic risk factors: A scientific statement from the National Lipid Association Nutrition and Lifestyle Task Force. *J Clin Lipidol* 2019;13(5):689-711.e1. doi: 10.1016/j.jacl.2019.08.003 PMID - 31611148

9. Ríos-Hoyo A, Gutiérrez-Salmeán G. New Dietary Supplements for Obesity: What We Currently Know. *Curr Obes Reports* 2016;5(2):262-70. doi: 10.1007/s13679-016-0214-y PMID - 27053066

10. He X, Duan Y, Yao K, et al. β-Hydroxy-β-methylbutyrate, mitochondrial biogenesis, and skeletal muscle health. *Amino Acids* 2016;48(3):653-64. doi: 10.1007/s00726-015-2126-7 PMID - 26573541

11. Landi F, Calvani R, Picca A, et al. Beta-hydroxy-beta-methylbutyrate and sarcopenia. *Curr Opin Clin Nutr* 2019;22(1):37-43. doi: 10.1097/mco.0000000000000524 PMID - 30489401

12. Peng LN, Cheng YC, Yu PC, et al. Oral Nutritional Supplement with β-hydroxy-β-methylbutyrate (HMB) Improves Nutrition, Physical Performance and Ameliorates Intramuscular Adiposity in Pre-Frail Older Adults: A Randomized Controlled Trial. *J Nutrition Heal Aging* 2021:1-7. doi: 10.1007/s12603-021-1621-7

13. Holeček M. Beta-hydroxy-beta-methylbutyrate supplementation and skeletal muscle in healthy and muscle - wasting conditions. *J Cachexia Sarcopenia Muscle* 2017;8(4):529-41. doi: 10.1002/jcsm.12208 PMID - 28493406

For peer review only - http://bmjopen.bmj.com/site/about/guidelines.xhtml

14. Cavallucci V, Pani G. The Leucine Catabolite and Dietary Supplement β-Hydroxy-β-Methyl Butyrate (HMB) as an Epigenetic Regulator in Muscle Progenitor Cells. *Metabolites* 2021;11(8):512. doi: 10.3390/metabo11080512 PMID - 34436453

15. Baxter JH, Carlos JL, Thurmond J, et al. Dietary toxicity of calcium β-hydroxy-β-methyl butyrate (CaHMB). *Food Chem Toxicol* 2005;43(12):1731-41. doi: 10.1016/j.fct.2005.05.016 PMID - 16006030

16. Nissen S, Sharp RL, Panton L, et al. β-Hydroxy-β-Methylbutyrate (HMB) Supplementation in Humans Is Safe and May Decrease Cardiovascular Risk Factors. *J Nutrition* 2000;130(8):1937-45. doi: 10.1093/jn/130.8.1937 PMID - 10917905

17. Rathmacher JA, Nissen S, Panton L, et al. Supplementation with a combination of beta-hydroxy-beta-methylbutyrate (HMB), arginine, and glutamine is safe and could improve hematological parameters. *Jpen-parenter Enter* 2004;28(2):65-75. doi: 10.1177/014860710402800265 PMID - 15080599

18. Wu H, Xia Y, Jiang J, et al. Effect of beta-hydroxy-beta-methylbutyrate supplementation on muscle loss in older adults: A systematic review and meta-analysis. *Arch Gerontol Geriat* 2015;61(2):168-75. doi: 10.1016/j.archger.2015.06.020 PMID - 26169182

19. Lin Z, Zhao Y, Chen Q. Effects of oral administration of β-hydroxy β-methylbutyrate on lean body mass in older adults: a systematic review and meta-analysis. *Eur Geriatr Med* 2021;12(2):239-51. doi: 10.1007/s41999-020-00409-9 PMID - 33034021

20. Panton LB, Rathmacher JA, Baier S, et al. Nutritional supplementation of the leucine metabolite β-hydroxy-β-methylbutyrate (hmb) during resistance training11Study funded by Metabolic Technologies, Inc. *Nutrition* 2000;16(9):734-39. doi: 10.1016/s0899-9007(00)00376-2 PMID - 10978853

21. Chen C, Lu FC, China DoDCMoHPR. The guidelines for prevention and control of overweight and obesity in Chinese adults. *Biomed Environ Sci Bes* 2004;17 Suppl:1-36.

22. Tremblay MS, Aubert S, Barnes JD, et al. Sedentary Behavior Research Network (SBRN) – Terminology Consensus Project process and outcome. *Int J Behav Nutr Phy* 2017;14(1):75. doi: 10.1186/s12966-017-0525-8 PMID - 28599680

23. Yoshimura E, Kumahara H, Tobina T, et al. Lifestyle Intervention Involving Calorie Restriction with or without Aerobic Exercise Training Improves Liver Fat in Adults with Visceral Adiposity. *J Obes* 2014;2014:1-8. doi: 10.1155/2014/197216 PMID - 24864199

24. China" GAoSo. Chinese National Physical Fitness Evaluation Standard (CNPFES). Beijing2003.

25. Molarius A, Seidell JC, Sans S, et al. Waist and hip circumferences, and waist-hip ratio in 19 populations of the WHO MONICA Project. *Int J Obesity* 1999;23(2):116-25. doi: 10.1038/sj.ijo.0800772 PMID - 10078844

26. Lee PH, Macfarlane DJ, Lam TH, et al. Validity of the international physical activity questionnaire short form (IPAQ-SF): A systematic review. *Int J Behav Nutr Phy* 2011;8(1):115. doi: 10.1186/1479-5868-8-115 PMID - 22018588

27. Bohannon RW. Sit-to-Stand Test for Measuring Performance of Lower Extremity Muscles. *Percept Motor Skill* 1994;80(1):163-66. doi: 10.2466/pms.1995.80.1.163 PMID - 7624188

28. Watanabe D, Nanri H, Sagayama H, et al. Estimation of Energy Intake by a Food Frequency Questionnaire: Calibration and Validation with the Doubly Labeled Water Method in Japanese Older People. *Nutrients* 2019;11(7):1546. doi: 10.3390/nu11071546 PMID - 31323937

For peer review only

29. Hollaender N, Gonzalez-Maffe J, Jehl V. Quantitative assessment of adverse events in clinical trials: Comparison of methods at an interim and the final analysis. *Biometrical J* 2020;62(3):658-69. doi: 10.1002/bimj.201800234 PMID - 31756032

30. Chen L-K, Woo J, Assantachai P, et al. Asian Working Group for Sarcopenia: 2019 Consensus Update on Sarcopenia Diagnosis and Treatment. *J Am Med Dir Assoc* 2020(Geriatr Gerontol Int 17 2017) doi: 10.1016/j.jamda.2019.12.012

31. Association WM. World Medical Association Declaration of Helsinki: Ethical Principles for Medical Research Involving Human Subjects. *Jama* 2013;310(20):2191-94. doi: 10.1001/jama.2013.281053 PMID - 24141714

32. Chan A-W, Tetzlaff JM, Gøtzsche PC, et al. SPIRIT 2013 explanation and elaboration: guidance for protocols of clinical trials. *Bmj Br Medical J* 2013;346(jan08 15):e7586. doi: 10.1136/bmj.e7586 PMID - 23303884

33. Albert FJ, Morente-Sánchez J, Ortega FB, et al. Usefulness of β-hydroxy-β-methylbutyrate (HMB) supplementation in different sports: an update and practical implications. *Nutrición Hosp* 2015;32(1):20-33. doi: 10.3305/nh.2015.32.1.9101 PMID - 26262692

34. Hashempour A, Hooshmand S, Tabesh MR, et al. Effect of 6-week HMB (beta-hydroxy-beta methylbutyrate) supplementation on muscle strength and body composition in sedentary overweight women. *Obes Medicine* 2019;15:100115. doi: 10.1016/j.obmed.2019.100115

35. Rondanelli M, Nichetti M, Peroni G, et al. Effect of a Food for Special Medical Purposes for Muscle Recovery, Consisting of Arginine, Glutamine and Beta-Hydroxy-Beta-Methylbutyrate on Body Composition and Skin Health in Overweight and Obese Class I Sedentary Postmenopausal Women. *Nutrients* 2021;13(3) doi: 10.3390/nu13030975

36. Hurt RT, Ebbert JO, Croghan I, et al. The Comparison of Segmental Multi-frequency BIA and DXA for Estimating Lean Body Mass and Percent Body Fat in an Ambulatory Population. *Jpen-parenter Enter* 2020 doi: 10.1002/jpen.1994 PMID - 32794583

Figure legends

Figure 1. Flow chart of this trial.

For peer review only

For peer review only - http://bmjopen.bmj.com/site/about/guidelines.xhtml

Table 1 Schedule of enrollment, interventions, and assessments

	Enrolment	Allocation	Post-allocation			
	Visit 1	Visit 2	Visit 3	Visit 4	Visit 5	Visit 6
Timepoint	Screening (-4 to 0 wks.)	Baseline (0 wk.)	3 wks. +/- 2 ds	6 wks. +/- 2 ds	9 wks. +/- 2 ds	12 wks. +/- 2 ds
**Enrollment**						
Consent	√					
Inclusion & exclusion	√					
Demographics	√					
Medical history	√					
Body height	√					
Bodyweight	√					
Blood pressure	√					
Heart rate	√					
BIA	√					
Allocation		√				
**Interventions**†						
HMB nutritional supplement + caloric restriction		●—	—	—	—	—●
Placebo + caloric restriction		●—	—	—	—	—●
**Assessments**						
Inclusion & exclusion		√				
Bodyweight		√		√		√
Blood pressure		√		√		√
Heart rate		√		√		√
WC		√		√		√
HC		√		√		√
IPAQ-SF		√	√	√	√	√
FFQ		√	√	√	√	√
BIA		√		√		√
Sit-to-stand test		√		√		√
HGS		√		√		√
Blood tests‡		√				√
Adverse events*			√	√	√	√
Adherence*			√	√	√	√

Abbreviations: BIA: bioimpedance analysis; FFQ: Food Frequency Questionnaire; HC: hip circumference; HGS: handgrip strength; IPAQ-SF: International Physical Activity Questionnaire Short Form; WC: waist circumference

† Both intervention groups will also receive dietary instruction.

‡ The blood tests will include glucose metabolism (fasting glucose and insulin), albumin, hemoglobin, alanine aminotransferase, aspartate aminotransferase, gamma-glutamyl transpeptidase, Cystatin C, creatinine, estimated glomerular filtration rate, lipid

For peer review only - http://bmjopen.bmj.com/site/about/guidelines.xhtml

profiles, and full blood counts.

* Weekly WeChat or telephone contacts will be performed to assess adverse events and adherence (including dietary compliance).

For peer review only

For peer review only - http://bmjopen.bmj.com/site/about/guidelines.xhtml

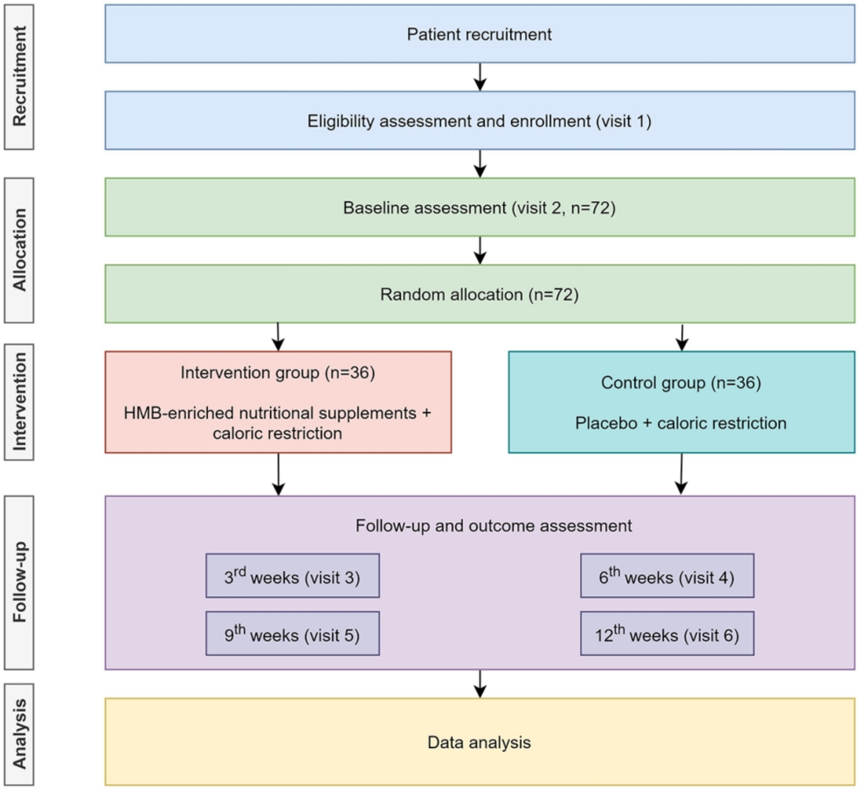

Figure 1. Flow chart of this trial.

Reporting checklist for protocol of a clinical trial.

Based on the SPIRIT guidelines.

Instructions to authors

Complete this checklist by entering the page numbers from your manuscript where readers will find each of the items listed below.

Your article may not currently address all the items on the checklist. Please modify your text to include the missing information. If you are certain that an item does not apply, please write "n/a" and provide a short explanation.

Upload your completed checklist as an extra file when you submit to a journal.

In your methods section, say that you used the SPIRITreporting guidelines, and cite them as:

Chan A-W, Tetzlaff JM, Gøtzsche PC, Altman DG, Mann H, Berlin J, Dickersin K, Hróbjartsson A, Schulz KF, Parulekar WR, Krleža-Jerić K, Laupacis A, Moher D. SPIRIT 2013 Explanation and Elaboration: Guidance for protocols of clinical trials. BMJ. 2013;346:e7586

		Reporting Item	Page Number
**Administrative information**			
Title	#1	Descriptive title identifying the study design, population, interventions, and, if applicable, trial acronym	1

For peer review only

Trial registration	#2a	Trial identifier and registry name. If not yet registered, name of intended registry	2
Trial registration: data set	#2b	All items from the World Health Organization Trial Registration Data Set	N/A
Protocol version	#3	Date and version identifier	16
Funding	#4	Sources and types of financial, material, and other support	17
Roles and responsibilities: contributorship	#5a	Names, affiliations, and roles of protocol contributors	16-17
Roles and responsibilities: sponsor contact information	#5b	Name and contact information for the trial sponsor	1
Roles and responsibilities: sponsor and funder	#5c	Role of study sponsor and funders, if any, in study design; collection, management, analysis, and interpretation of data; writing of the report; and the decision to submit the report for publication, including whether they will have ultimate authority over any of these activities	17
Roles and responsibilities: committees	#5d	Composition, roles, and responsibilities of the coordinating centre, steering committee, endpoint adjudication committee, data management team, and	N/A

For peer review only

		other individuals or groups overseeing the trial, if applicable (see Item 21a for data monitoring committee)	
**Introduction**			
Background and rationale	#6a	Description of research question and justification for undertaking the trial, including summary of relevant studies (published and unpublished) examining benefits and harms for each intervention	3-4
Background and rationale: choice of comparators	#6b	Explanation for choice of comparators	4
Objectives	#7	Specific objectives or hypotheses	4
Trial design	#8	Description of trial design including type of trial (eg, parallel group, crossover, factorial, single group), allocation ratio, and framework (eg, superiority, equivalence, non-inferiority, exploratory)	5
**Methods: Participants, interventions, and outcomes**			
Study setting	#9	Description of study settings (eg, community clinic, academic hospital) and list of countries where data will be collected. Reference to where list of study sites can be obtained	5

For peer review only

Eligibility criteria	#10	Inclusion and exclusion criteria for participants. If applicable, eligibility criteria for study centres and individuals who will perform the interventions (eg, surgeons, psychotherapists)	5-7
Interventions: description	#11a	Interventions for each group with sufficient detail to allow replication, including how and when they will be administered	8
Interventions: modifications	#11b	Criteria for discontinuing or modifying allocated interventions for a given trial participant (eg, drug dose change in response to harms, participant request, or improving / worsening disease)	7
Interventions: adherance	#11c	Strategies to improve adherence to intervention protocols, and any procedures for monitoring adherence (eg, drug tablet return; laboratory tests)	8
Interventions: concomitant care	#11d	Relevant concomitant care and interventions that are permitted or prohibited during the trial	8
Outcomes	#12	Primary, secondary, and other outcomes, including the specific measurement variable (eg, systolic blood pressure), analysis metric (eg, change from baseline, final value, time to event), method of aggregation (eg, median, proportion), and time point for each outcome. Explanation of the clinical relevance of chosen efficacy and harm outcomes is strongly recommended	8-12

For peer review only

Participant timeline	#13	Time schedule of enrolment, interventions (including any run-ins and washouts), assessments, and visits for participants. A schematic diagram is highly recommended (see Figure)	12 , Table1
Sample size	#14	Estimated number of participants needed to achieve study objectives and how it was determined, including clinical and statistical assumptions supporting any sample size calculations	12-13
Recruitment	#15	Strategies for achieving adequate participant enrolment to reach target sample size	5
**Methods: Assignment of interventions (for controlled trials)**			
Allocation: sequence generation	#16a	Method of generating the allocation sequence (eg, computer-generated random numbers), and list of any factors for stratification. To reduce predictability of a random sequence, details of any planned restriction (eg, blocking) should be provided in a separate document that is unavailable to those who enrol participants or assign interventions	7
Allocation concealment mechanism	#16b	Mechanism of implementing the allocation sequence (eg, central telephone; sequentially numbered, opaque, sealed envelopes), describing any steps to conceal the sequence until interventions are assigned	7

For peer review only

Allocation: implementation	#16c	Who will generate the allocation sequence, who will enrol participants, and who will assign participants to interventions	7
Blinding (masking)	#17a	Who will be blinded after assignment to interventions (eg, trial participants, care providers, outcome assessors, data analysts), and how	8
Blinding (masking): emergency unblinding	#17b	If blinded, circumstances under which unblinding is permissible, and procedure for revealing a participant's allocated intervention during the trial	8
**Methods: Data collection, management, and analysis**			
Data collection plan	#18a	Plans for assessment and collection of outcome, baseline, and other trial data, including any related processes to promote data quality (eg, duplicate measurements, training of assessors) and a description of study instruments (eg, questionnaires, laboratory tests) along with their reliability and validity, if known. Reference to where data collection forms can be found, if not in the protocol	9
Data collection plan: retention	#18b	Plans to promote participant retention and complete follow-up, including list of any outcome data to be	9

For peer review only

		collected for participants who discontinue or deviate from intervention protocols	
Data management	#19	Plans for data entry, coding, security, and storage, including any related processes to promote data quality (eg, double data entry; range checks for data values). Reference to where details of data management procedures can be found, if not in the protocol	12
Statistics: outcomes	#20a	Statistical methods for analysing primary and secondary outcomes. Reference to where other details of the statistical analysis plan can be found, if not in the protocol	13-14
Statistics: additional analyses	#20b	Methods for any additional analyses (eg, subgroup and adjusted analyses)	14
Statistics: analysis population and missing data	#20c	Definition of analysis population relating to protocol non-adherence (eg, as randomised analysis), and any statistical methods to handle missing data (eg, multiple imputation)	13
**Methods: Monitoring**			
Data monitoring: formal committee	#21a	Composition of data monitoring committee (DMC); summary of its role and reporting structure; statement of whether it is independent from the sponsor and competing interests; and reference to where further details about its charter can be found, if not in the	12

For peer review only

		protocol. Alternatively, an explanation of why a DMC is not needed	
Data monitoring: interim analysis	#21b	Description of any interim analyses and stopping guidelines, including who will have access to these interim results and make the final decision to terminate the trial	12
Harms	#22	Plans for collecting, assessing, reporting, and managing solicited and spontaneously reported adverse events and other unintended effects of trial interventions or trial conduct	11
Auditing	#23	Frequency and procedures for auditing trial conduct, if any, and whether the process will be independent from investigators and the sponsor	N/A
**Ethics and dissemination**			
Research ethics approval	#24	Plans for seeking research ethics committee / institutional review board (REC / IRB) approval	14
Protocol amendments	#25	Plans for communicating important protocol modifications (eg, changes to eligibility criteria, outcomes, analyses) to relevant parties (eg, investigators, REC / IRBs, trial participants, trial registries, journals, regulators)	14

For peer review only

Consent or assent	#26a	Who will obtain informed consent or assent from potential trial participants or authorised surrogates, and how (see Item 32)	14
Consent or assent: ancillary studies	#26b	Additional consent provisions for collection and use of participant data and biological specimens in ancillary studies, if applicable	N/A
Confidentiality	#27	How personal information about potential and enrolled participants will be collected, shared, and maintained in order to protect confidentiality before, during, and after the trial	12
Declaration of interests	#28	Financial and other competing interests for principal investigators for the overall trial and each study site	17
Data access	#29	Statement of who will have access to the final trial dataset, and disclosure of contractual agreements that limit such access for investigators	18
Ancillary and post trial care	#30	Provisions, if any, for ancillary and post-trial care, and for compensation to those who suffer harm from trial participation	N/A
Dissemination policy: trial results	#31a	Plans for investigators and sponsor to communicate trial results to participants, healthcare professionals, the public, and other relevant groups (eg, via publication, reporting in results databases, or other data sharing arrangements), including any publication restrictions	14

For peer review only

Dissemination policy: authorship	#31b	Authorship eligibility guidelines and any intended use of professional writers	N/A
Dissemination policy: reproducible research	#31c	Plans, if any, for granting public access to the full protocol, participant-level dataset, and statistical code	N/A

Appendices

Informed consent materials	#32	Model consent form and other related documentation given to participants and authorised surrogates	Supplementary file
Biological specimens	#33	Plans for collection, laboratory evaluation, and storage of biological specimens for genetic or molecular analysis in the current trial and for future use in ancillary studies, if applicable	N/A

The SPIRIT Explanation and Elaboration paper is distributed under the terms of the Creative Commons Attribution License CC-BY-NC. This checklist was completed on 12. July 2021 using https://www.goodreports.org/, a tool made by the EQUATOR Network in collaboration with Penelope.ai

For peer review only
